# Adaptive differentiation coincides with local bioclimatic conditions along an elevational cline in populations of a lichen-forming fungus

**DOI:** 10.1186/s12862-017-0929-8

**Published:** 2017-03-31

**Authors:** Francesco Dal Grande, Rahul Sharma, Anjuli Meiser, Gregor Rolshausen, Burkhard Büdel, Bagdevi Mishra, Marco Thines, Jürgen Otte, Markus Pfenninger, Imke Schmitt

**Affiliations:** 1 Senckenberg Biodiversity and Climate Research Centre (BiK-F), Senckenberganlage 25, 60325 Frankfurt am Main, Germany; 2 Institut für Ökologie, Evolution und Diversität, Goethe-Universität Frankfurt, Max-von-Laue-Str. 9, 60438 Frankfurt am Main, Germany; 3 Plant Ecology and Systematics, Biology Department, University of Kaiserslautern, 67653 Kaiserslautern, Germany

**Keywords:** Adaptation, Altitudinal, Climate change, Fungi, Pool-Seq, ﻿Population genomics, Symbiosis, SNP, Gradient

## Abstract

**Background:**

Many fungal species occur across a variety of habitats. Particularly lichens, fungi forming symbioses with photosynthetic partners, have evolved remarkable tolerances for environmental extremes. Despite their ecological importance and ubiquity, little is known about the genetic basis of adaption in lichen populations. Here we studied patterns of genome-wide differentiation in the lichen-forming fungus *Lasallia pustulata* along an altitudinal gradient in the Mediterranean region. We resequenced six populations as pools and identified highly differentiated genomic regions. We then detected gene-environment correlations while controlling for shared population history and pooled sequencing bias, and performed ecophysiological experiments to assess fitness differences of individuals from different environments.

**Results:**

We detected two strongly differentiated genetic clusters linked to Mediterranean and temperate-oceanic climate, and an admixture zone, which coincided with the transition between the two bioclimates. High altitude individuals showed ecophysiological adaptations to wetter and more shaded conditions. Highly differentiated genome regions contained a number of genes associated with stress response, local environmental adaptation, and sexual reproduction.

**Conclusions:**

Taken together our results provide evidence for a complex interplay between demographic history and spatially varying selection acting on a number of key biological processes, suggesting a scenario of ecological speciation.

**Electronic supplementary material:**

The online version of this article (doi:10.1186/s12862-017-0929-8) contains supplementary material, which is available to authorized users.

## Background

Fungi are diverse and ubiquitous, having evolved over time to occupy a wide range of ecological niches. Some fungal species are exceptionally proficient at surviving a broad range of environmental conditions. In nature, these species can inhabit latitudinal and altitudinal clines that span considerable temperature ranges [1]. Individuals from species with broad ecological amplitudes exhibit local adaptation when divergent selection is strong relative to the rate of gene flow [2, 3]. Locally adapted individuals show higher fitness in their focal environment relative to immigrants. Despite the wealth of studies investigating genetic structure and dispersal of fungi, the processes that shape adaptive genetic polymorphism in wild populations are not well understood.

Environmental factors have been shown to be drivers of local adaptation in diverse fungi. For example, temperature differences are responsible for the maintenance of differentially adapted populations of pathogens [4]. Temperature changes were also shown to be drivers of adaptation in natural populations of the saprotrophic fungus *Neurospora crassa*, resulting in genomic islands of differentiation involved in cold-response and circadian rhythm [5]. Differences in climate and soil salinity correlated with regions of extreme genomic divergence between coastal and montane populations of an ectomycorrhizal basidiomycete [6]. Furthermore, experimental evidence suggests that salinity and temperature are drivers of ecological isolation in experimentally derived lineages of baker’s yeast *Saccharomyces cerevisiae* [7]. In some cases, ecological divergence can promote reproductive barriers between populations of differentially adapted ecotypes [8], as is the case in the filamentous fungus *Neurospora crassa* [9]. Overall however, adaptive genomic polymorphisms have been investigated only in a small set of fungal species and life styles.

Lichen-forming fungi, which constitute about half of the described ascomycetes, are a nutritionally specialized group of fungi that form obligate symbiotic associations with green algae and bacteria [10]. Lichens are suitable to study local adaptation because they can tolerate extreme environmental conditions and sustain growth despite frequent cycles of desiccation and rehydration, low nutrient availability, and large fluctuations in temperature (e.g., [11, 12]). The distributional ranges of many lichens span broad climatic ranges [13–16]. Furthermore, long-lived, sessile organisms such as lichens experience strong selection pressures [17]. This may lead to reduced survival of maladapted individuals, and create steeper genetic gradients between differentially selected populations [18]. Environmental stressors, such as drought and high-light conditions, have been shown to trigger physiological adjustments in different lichens [19–21]. The genetic bases of these adaptive responses are currently poorly understood. Many species of lichenized fungi show genetic differentiation among populations despite ongoing gene flow, even across thousands of kilometers. This suggests a role of spatially varying selection in maintaining biogeographic structure (reviewed in [22]). Interestingly, one study reported gene pool associations with altitude and interpreted this as evidence for climate-driven local adaptation [23]. However, no study so far has specifically addressed adaptive diversity in geographically close (<20 km), but ecologically distant lichen populations.

Altitudinal gradients are suited to study local adaptation because ecological transitions are typically steep and occur at relatively short distances, thus limiting the confounding effect of distinct regional evolutionary histories [24]. Moreover, altitudinal gradients are also climatic gradients, characterized by decreasing temperature and atmospheric pressure, increasing relative air humidity, rainfall, and solar radiation with increasing altitude [25]. Thus, adaptation along altitudinal gradients can be explored as a proxy model for genomic responses to climate change [26–29].

Here we report on the population genomics of a lichen-forming ascomycete along an altitudinal gradient in the Mediterranean region. As model, we chose *Lasallia pustulata* (Umbilicariaceae), a species with a distribution from southern Europe to northern Scandinavia, which forms dense populations on exposed, siliceous rocks [30]. Using genomic data from geographically close populations along a steep altitudinal gradient in northern Sardinia (Italy), we analyzed whether genetic clusters were present, and whether relatedness between clusters was correlated with signatures of local adaptation. Heat, drought, and radiation stress constitute determining factors in the composition of biological communities inhabiting rocky outcrops and boulders in Mediterranean mountains [31]. Therefore we tested the hypothesis that environmental factors shape genome-wide population differentiation in lichenized fungi which occur across different bioclimatic regions. Specifically, we addressed the following questions: i) what is the genome-wide population structure and connectivity between geographically close populations along an elevation gradient?, ii) what are putative functions of highly differentiated genes between the genetic clusters?, iii) what are putative functions of the genes showing strong correlation with local climatic factors?, and iv) do individuals belonging to different genetic clusters (and environments) display fitness differences?

## Methods

### Study organism, study site, and sampling

**Fig. 1:**
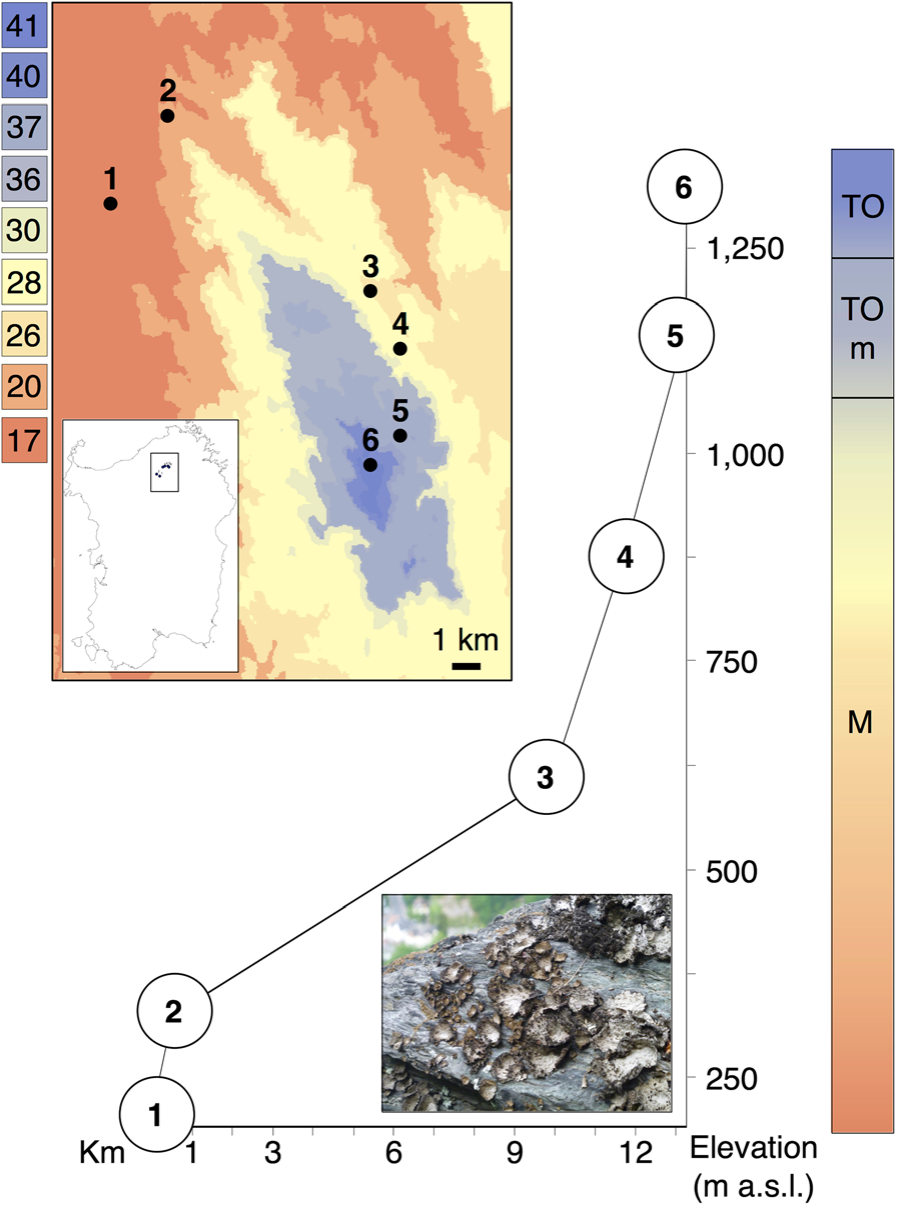
Location of the study site in north-eastern Sardinia (Italy) and location of six populations of *L. pustulata* along the elevation cline. The numbers in the color legend refer to the bioclimatic zones by [32]: 17 - lower mesomediterranean-upper dry-weak euoceanic, 20 - lower mesomediterranean-lower sub-humid-weak euoceanic, 26 - upper mesomediterranean-lower sub-humid-weak euoceanic, 28 - upper mesomediterranean-upper sub-humid-weak euoceanic, 30 - upper mesomediterranean-lower humid-weak euoceanic, 36 - upper mesotemperate (sub-Mediterranean)-lower humid-weak euoceanic, 37 - upper mesotemperate (sub-Mediterranean)-lower humid-weak semi-continental, 40 - lower supratemperate-lower humid-weak semi-continental, 41 - lower supratemperate-lower hyperhumid-weak semi-continental. The color gradient represents the bioclimatic profile of the cline, ranging from Mediterranean pluviseasonal oceanic (M) (populations 1–4) to temperate oceanic, submediterranean variant (TOm) (population 5), to temperate oceanic (TO) (population 6) climate

*Lasallia pustulata* is a foliose, rock-inhabiting, haploid lichen-forming ascomycete. Individuals are attached to the substrate with a central holdfast. *L. pustulata* has a mixed strategy of asexual and sexual cycles. Asexual reproduction via isidia – macroscopic dispersal units containing both symbionts that break off the mother thallus – is the typical (and in most populations only) way of reproduction. Isidia are considered to be detached from the thallus mainly by raindrops, and dispersed over short distances [30]. We collected samples from six populations along an altitudinal gradient in the Limbara massif (Sardinia, Italy). The transect extended from Lake Coghinas (population 1: 176 m a.s.l.) to Punta Balestrieri (population 6: 1303 m a.s.l.) covering a linear distance of ~13.5 km. Intermediate populations were located at 297 m a.s.l. (population 2), 588 m a.s.l. (population 3), 842 m a.s.l. (population 4), and 1125 m a.s.l. (population 5). The maximal linear distance between populations was ~9 km (Fig. 1). Populations were located on horizontal or gently sloping, fully sun-exposed rock faces in scattered Paleozoic granitic outcrops, and covered an area of ~50 m^2^. For each population, we collected 100 thallus pieces of ~8 mm in diameter. Our sampling design aimed at capturing the maximal diversity present at the sites. The minimum distance between sampled individuals was 50 cm to maximize the inclusion of different genets. Samples were collected with sterile tools and transferred into sterile 2 ml tubes.

The study area encompasses three distinct bioclimates: i) Mediterranean pluviseasonal oceanic (populations 1–4), ii) temperate oceanic submediterranean (population 5), and iii) temperate oceanic (population 6) (Fig. 1) [32]. Temperature data collected between May 28th 2014 and June 2nd 2015 from loggers positioned at the level of *L. pustulata* thalli (2 loggers per population) indicate that localities 1 to 4 have higher summer temperatures and are less prone to freezing during winter than populations 5 and 6. Logger data also showed that temperatures of the rock surfaces to which thalli are attached frequently exceeded 50 °C in summer. The populations therefore experienced seasonal temperature fluctuations on the order of >40 °C (see Additional file 1).

### DNA extraction and genome resequencing

For each population, we extracted genomic DNA separately from each individual using a CTAB-based method [33]. DNA concentration was measured with a Qubit fluorometer (dsDNA BR, Invitrogen). A pooled sample was created for each population containing equal amounts of DNA from each sample (Pool-seq). Library preparation (200–300 bp insert size), sequencing on an Illumina HiSeq2000 with 100 bp paired-end chemistry at ~90x coverage per population, as well as tags and adaptor removal were performed by GenXPro GmbH (Frankfurt am Main, Germany).

### Genome annotation

As reference genome, we used the draft assembly of *L. pustulata* available at the European Nucleotide Archive (http://www.ebi.ac.uk/ena/data/view/GCA_000938525.1). The draft genome is composed of 3891 scaffolds (average length of 10Kbp) for a total length of 39.2 Mbp and an N50 scaffold of size 104.3Kbp. The genome is ~92% complete according to an assessment with the software BUSCO 2.0 [34] and a lineage-specific set of Ascomycota single-copy orthologs.

For gene model prediction we used both ab-initio based methods and RNA-Seq derived transcript mapping onto the assembled genome following the method described in [35]. For this purpose, total RNA was isolated from a thallus of *L. pustulata* collected near Orscholz (Saarland, Germany; N49.5012, E6.5440) in July 2013 using the method by [36], and purified using the RNeasy MinElute Clean-up Kit (Qiagen). Paired-end sequencing was performed using Illumina MiSeq (2x250 bp) by StarSEQ (Mainz, Germany). RNA-Seq data was quality-filtered using Trimmomatic [37], with a length cutoff of 200 and a quality cutoff of 20 in a window of 5 bp.

We used Blast2GO [38] to annotate the predicted protein sequences with gene ontology (GO) terms and protein names using NCBI's *nr* database at an E-value cut-off of 1x10^−3^, and default weighting parameters. We also annotated each protein with InterPro domains using InterProScan [39].

### SNP analysis

We filtered out reads shorter than 80 bp, reads with N's, and reads with average base quality scores less than 26 along with their pairs using FastQFS [40]. Trimmed paired-end reads of each pool were mapped to the *L. pustulata* genome using BWA-MEM [41] and default parameters. Unambiguously aligned reads with a minimum mapping quality of 20 were extracted with SAMtools v1.18 [42]. Reads were sorted and duplicates were marked with Picard using the tools *SortSam.jar* and *MarkDuplicates.jar*. Single nucleotide polymorphisms were called with SAMtools (mpileup, [43]). Indels were detected and masked with PoPoolation [44] using the scripts *identify-genomic-indel-regions.pl* (−−min-count 2 −−indel-window 5) and *filter-pileup-by-gtf.pl*. The synchronized file was converted into a gene-based synchronized file using the script *create-genewise-sync.pl* in PoPoolation2 [45]. The coverage for each population was reduced to a uniform coverage of 30 with PoPoolation2 using the sync-file and the script *subsample-synchronized.pl* (−−without-replacement).

### Population genetics analyses

To characterize genome-wide patterns of variation, we estimated three population genetic parameters by accounting for pooling: i) *π,* a measure of polymorphism in a sample of sequences scaled to their length, ii) Watterson's θ (θ_W_), a measure of the number of segregating sites, and iii) Tajima's *D,* a measure of the skew of allele frequency distribution. All estimates were calculated in non-overlapping 10-kb windows across the genome using PoPoolation [44], assuming a minimum count of two. Differences in genetic diversity among populations were tested using linear mixed effect models in R 3.2.2 [46, 47]. For each diversity measure, models included population as a fixed effect predictor and incorporated scaffolds as a random effect across populations. Pairwise population comparisons were then obtained from post hoc Tukey contrasts of the respective model predictors [48].

To identify strongly differentiated alleles, we adopted an empirical outlier approach. Genetic differentiation (F_ST_) was calculated with *fst-sliding.pl* in PoPoolation2. We only considered SNPs with a minimum count of 4, a minimum quality of 20, and falling into the upper 0.5% tail of the F_ST_ distribution, corresponding to an F_ST_ threshold of 1.0. Highly differentiated SNPs were further inspected with Fisher’s exact test [49] to identify significant allele frequencies differences between population pairs using the script *fisher-test.pl* in PoPoolation2 and a Bonferroni-corrected *p*-value of 0.003. In addition, we estimated average F_ST_ across all polymorphic SNPs for each gene and only considered those falling into the upper 5% tail of distribution to be truly differentiated. Based on this analysis, we calculated the percentage of overlap between SNP-based and gene-based lists. SNPs were classified as genic and non-genic loci. Genic SNPs were further classified as exonic, coding, and intronic. Non-genic SNPs located in the 600 bp 5'-flanking sequence of each gene were considered putative promoter SNPs.

To visualize groups of populations with varying degrees of similarity to one another, we first obtained a reduced set of pairwise F_ST_ distance matrices based on sample quantiles (0.975, 0.75, 0.5, 0.25, 0.025) of the full set of distances across all polymorphic SNPs. The resulting set of pairwise (quantile) distances was then jointly analyzed using a three-way generalization of classical multidimensional scaling (DISTATIS, [50]). DISTATIS calculates a compromise distance space from the weighted average of all cross-product matrices derived from the set of quantile distance matrices. This compromise can then be used to visualize positional relations among populations 1 – 6 based on their genetic distances. Moreover, we obtained 95% confidence intervals around each population's compromise position using bootstrap resampling [51].

To reconstruct the historical relationships among populations using their current genome-wide allele frequencies, we used TreeMix v1.12 [52]. We created a maximum likelihood phylogeny of the populations based on all polymorphic SNPs, using blocks of 500 SNPs to account for linkage disequilibrium. To test for the presence of admixture and migration among populations, we calculated *f3* and *f4* statistics. These statistics are formal tests for admixture as they detect correlations in allele frequencies that are not compatible with population evolution following a bifurcating tree. To calculate *f3* and *f4* statistics we used the *threepop* and *fourpop* functions in TreeMix on all possible triplets and tetraplets population groups. To further explore population relationships, we calculated neighbor joining trees for the correlation matrix obtained with Bayenv2.0 (see below) and for the matrix of pairwise F_ST_ values calculated across all polymorphic sites using the package *ape* in R [46].

To corroborate the hypothesis of admixture, we estimated gene flow among the three major groups (A: populations 1 to 4, B: population 5, C: population 6) across multiple intergenic polymorphic loci using the coalescent-based method MIGRATE-N 3.2.6 [53]. We estimated relative effective population size (N_e_) according to the relation θ = N_e_μ, assuming identical but unknown mutation rates (μ) in all populations. Haplotypes (20 per population) were obtained by parsing the output files of PoPoolation2 for genomic regions shorter than 90 bp, and containing three or more SNPs. To minimize the chance that the loci are linked, we retained only one such region per scaffold. This information was then used to extract the individual Illumina reads covering this region from the mapping file for each population. The reads were aligned and trimmed to the informative region. The alignments were filtered for a minimum coverage of 15x, maximum coverage of 100x and minimum length of 20 bp in all populations. This resulted in a data set of 5880 sequences from 49 loci, covering in total 1763 bp. All previously described steps were performed with custom Python scripts (see [54]). Bayesian estimates of number of migrants (Nm) and θ were obtained under an unconstrained migration model with variable θ using MIGRATE-N 3.2.6 [53] for each pair of genetic clusters separately. We used a uniform prior on both θ (0.0-0.40) and Nm (0.0-600). A Metropolis-coupled Monte-Carlo chain with static heating (1.0, 1.5, 3, 1 × 10^6^) was run for 1.8 × 10^6^ generations, recording every 600th step after a burn-in period of 6 × 10^4^ generations. Convergence was monitored with Tracer (http://beast.bio.ed.ac.uk/). All effective sample sizes of the MCMC chain were larger than 10^4^.

### Environmental association analysis

To identify candidate loci for altitude specific adaptation, we correlated allele frequencies of populations with the environment using Bayenv2.0 [55]. Bayenv2.0 models the sampling error of pooled sequencing, and accounts for the confounding effect of neutral, demographic signals. To summarize the climate along the cline, we used elevation and 19 bioclimatic variables from the WorldClim database [56]. Correlation among these was checked using the function *rcorr* in R [46]. Elevation was strongly correlated with all bioclimatic variables. Variation in the bioclimatic variables and elevation was thus summarized using PCA, resulting in one composite climate variable explaining 97.6% of the variance, hereafter referred to as *env1* (see Additional file 2). To build the reference covariance matrix of population allele frequencies, we used a subsample of 10,000 polymorphic SNPs based on 1,000,000 MCMC iterations. To ensure convergence we estimated a second matrix from another subsample of 10,000 SNPs. We explored gene-environment correlation by estimating the statistic *Z* between allele frequencies of all SNPs and *env1* per population for 200,000 iterations. SNPs with the highest score possible for *Z* (i.e., *Z* = 0.5) were considered as showing strong support for a non-zero correlation.

### Gene ontology enrichment

Gene Ontology (GO) term enrichment is a technique for interpreting the functions of a set of genes making use of the GO system of classification (http://www.geneontology.org/). In this system genes are assigned to predefined bins depending on their functional characteristics in a species-independent manner. An enrichment analysis will find which GO terms are over-represented in a given data set using the annotations for that gene set. We used the R package *topGO* [57] to search for an enrichment of different GO categories. The analysis was performed for i) the set of genes containing SNPs falling into the upper 0.5% tail of the SNP-derived F_ST_ distribution, ii) the subset of these that are differentially fixed between the low altitude (population 1 to 4) and high altitude (population 6) genetic clusters, and iii) the set of genes containing the Baynev2.0 top 1% SNPs. All genes with a GO annotation were used as background. Significance for each GO-identifier was computed with Fisher’s exact test at α = 5%. We used the ‘elim’ method in topGO to iteratively remove genes mapped to significant GO terms from more general terms, thus reducing the rate of false positives. Only GOs with more than three associated genes were considered. We used the REVIGO tool [58] to produce summaries of non-redundant GO terms grouped into functional categories.

### Ecophysiology

We performed ecophysiological experiments to assess differences in physiological traits in the populations. Measurements were performed on three samples per population. For this analysis, we randomly selected specimens with a minimum diameter of 6 cm to have enough material to perform replicate measurements. To explore the genetic relatedness of the individuals we genotyped each specimen at six loci, covering a total of approximately 4.1 Kbp. We selected three of the loci from genes containing top 0.5% differentiated SNPs, and three from those with top Bayenv2.0 SNPs. For primers and genetic characteristics of the loci see Additional file 3.

We investigated the thalli for differences in biomass and chlorophyll content per surface area. To calculate the specific thallus area (mm^2^/mg), we first determined the thallus size by photographing wetted thalli on scale paper using a binocular microscope and the AxioVision software (Carl Zeiss, Jena, Germany). We determined the dry weights (DW) of these thalli by weighing after 3 days of oven drying at 60 °C. We also measured thallus chlorophyll content according to [59]. Statistical significance of differences in biomass and chlorophyll content between groups was determined using a Mann–Whitney test.

To characterize the physiological response to different light conditions, and different thallus water contents, we conducted CO_2_ gas exchange measurements using a portable mini cuvette system (GFS 3000, Walz Company, Effeltrich, Germany). We measured the response of net photosynthesis (NP) and dark respiration (DR) to thallus water content (WC) for a subsample of six thalli, representing the two main genetic groups present along the gradient. We measured complete desiccation cycles (from water saturated to air dry thalli) at saturating light (750 μmol photons m^−2^s^−1^), ambient CO_2_, at 17 °C (within the optimal temperature range for CO_2_-gas exchange of this species). We weighed the samples between each measurement and later extrapolated WC as a percentage of DW. We determined DW after 5 days in a desiccator over silica gel. We considered ninety percent of maximum NP to be a reasonable estimate for optimal water saturation. We measured the samples at 3–15 h intervals for NP and DR. Immediately after each measurement we removed the samples from the cuvettes and determined their weight to calculate the decrease in WC. This process was continued for 97 h with each sample until CO_2_ exchange ceased due to complete drying. Statistical significance of differences in TWC and maximum NP between groups was determined using a *t*-test.

## Results

### Reference gene set

We identified a total of 8268 genes in the *L. pustulata* genome. In total 5747 genes were assigned a GO term.

### Genome-wide variation

After adapter and quality trimming, we obtained 179,809,145 paired-end reads (26.6–32.1 million per pool, total of 16.2 GB, average coverage per pool: 89.91x).

To examine sequence variation, we used two estimates of nucleotide diversity, *π* and θ_W_. When averaged for all 10-kb windows across the genome, estimates of *π* were highest in population 5 (*π* = 0.006 ± 0.004), and lowest in population 4 (*π* = 0.004 ± 0.003). Estimates of θ_W_ were instead highest in population 2 (θ_W_ = 0.005 ± 0.003), and lowest in population 4 (θ_W_ = 0.003 ± 0.003). To examine deviation from neutrality, we calculated Tajima's *D* in 10-kb windows across the genome. Average *D* deviated from neutrality and differed significantly among populations. *D* was negative in populations 2 (*D* = −0.839 ± 0.713) and 6 (*D* = −0.260 ± 0.322), and positive in all other populations, being highest in population 5 (*D* = 1.112 ± 0.54) (Table 1, Additional file 4, Additional file 5).

**Table 1:** Sampling locations of *Lasallia pustulata* populations and the mean and standard deviation for three standard population genetic parameters, *π*, Watterson's θ (θ_W_), and Tajima's *D* in nonoverlapping 10-kb windows across the genome of *L. pustulata*

Population	Lat	Long	Altitude (m)	nucleotide diversity π (SE)	Watterson's θ (SE)	Tajima's *D* (SE)
1	40.7577	9.0794	176	0.00375 (0.00349)	0.00355 (0.00333)	0.15417 (0.82987)
2	40.7778	9.0546	297	0.00441 (0.00335)	0.00536 (0.00322)	−0.83941 (0.71274)
3	40.8503	9.1119	588	0.00368 (0.00337)	0.00358 (0.00332)	0.04974 (0.926)
4	40.8568	9.1340	842	0.00359 (0.00333)	0.00334 (0.00315)	0.19892 (0.96759)
5	40.8573	9.1642	1125	0.00551 (0.00386)	0.00439 (0.00366)	1.11196 (0.53974)
6	40.8524	9.1732	1303	0.004121 (0.00349)	0.00444 (0.00373)	−0.26011 (0.32173)

### Patterns of genetic differentiation

**Fig. 2:**
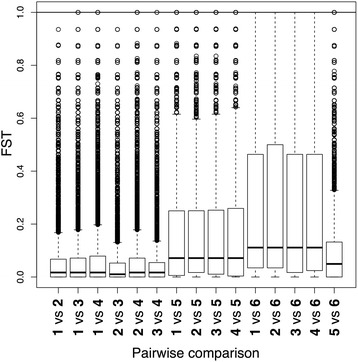
Pairwise F_ST_ comparisons of populations 1 to 6 across a total of 722,401 SNPs. *Boxes* extend from the first to the third quartiles, with a *horizontal line* indicating the median. The horizontal line across the graph indicates the top 0.5 quantile (F_ST_ = 1)

Among the six populations we identified 722,401 polymorphic SNPs (Table 2). Mean pairwise F_ST_ based on all polymorphic sites was moderate with an average of 0.124, and ranging from 0.044 (Pool1 vs. Pool4) to 0.236 (Pool2 vs. Pool6) (Fig. 2).

**Table 2:** Number of variants for all annotated features of the *L. pustulata* genome

Feature	All SNPS	top *Z* Bayenv2.0	top 0.5% F_ST_	top 0.5% F_ST_ fixed A-C^a^
GENIC	230,032	1171	11,492	1308
exon	3125	22	134	8
CDS	173,785	853	8358	968
intron	53,122	296	3000	332
INTERGENIC	492,369	1807	19,079	2862
promoters	40,460	19	15	7
Total SNPs	722,401	2978	30,571	4170
Genes	7267	616	2944	595

^a^A (populations 1 to 4), C (population 6)

A total of 30,571 SNPs located in 2944 genes fell into the upper 0.5% tail of the distribution. Of these, 4170 SNPs in 595 genes were differentially fixed between the low (1 to 4) and high-altitude (6) population clusters. When calculating average F_ST_ across all polymorphic sites within a given gene, 2413 genes fell into the top 5% tail. Our SNP-based approach detected 72.9% of the highly differentiated genes.

**Fig. 3:**
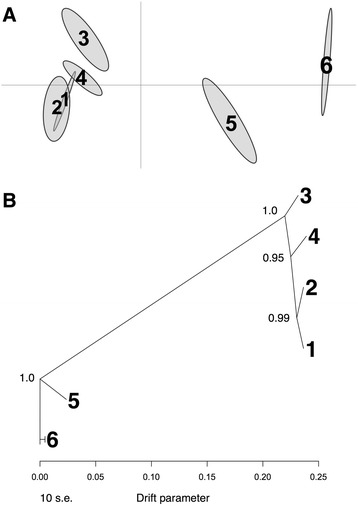
**a** Compromise configuration of categories between populations based on the F_ST_ quantile distance matrix for 722,401 polymorphic SNPs, with 95% tolerance ellipses. **b** Tree inferred with TreeMix for the six population of *L. pustulata* based on 722,401 polymorphic SNPs. Numbers at the branching points are support values from bootstrapping based on 1000 runs

We found strong genetic structure separating lower altitude populations (populations 1 to 4) from the other populations. The multidimensional scaling of the F_ST_ quantile distance SNP matrix illustrated the close genetic affinities of populations 1 to 4, with population 5 occupying an intermediate position between these and population 6 (Fig. 3a). The tree-based analyses showed a similar, well-resolved structure (Fig. 3b), with the tree derived from the Bayenv2.0 correlation matrix showing longer internal branches (see Additional file 6). Bayenv2.0- and F_ST_-matrices were highly correlated (Mantel-test, *r* = 0.999, *P* = 0.001; see Additional file 7).

Using the *threepop* test we found clear evidence of admixture at the level of population 5. All population triplets having population 5 as the admixed group displayed significantly negative *f3* values (see Additional file 8). This is in accord with the higher nucleotide diversity and positive Tajima's *D* values for population 5 (see Additional file 4). We also found support for migration among populations as 35 out of 45 four-population tests rejected all possible tree topologies without migration (|z| > 3, i.e., *p* < 0.001). We inferred higher significance for pairs grouping together population 5 and 6 with one of the lower altitude populations, respectively (see Additional file 8), which is also in accordance with the proposed admixture scenario.

To further describe the migration pattern, we performed estimations of migration rates among the major genetic groups with MIGRATE-N 3.2.6. Mutation-scaled effective population sizes varied between groups, ranging from θ = ~0.054 in group A (pop. 1–4) to θ = ~0.065 in group B (pop. 5) (see Additional file 9). Migration rates varied by several orders of magnitude. Results supported the hypothesis that the genetic diversity of population 5 is the result of admixture from the other two genetic groups, while gene flow rates between the other groups are negligible in comparison. This is in line with the F_ST_- and tree-based analyses.

### Candidate loci for local adaptation

To identify candidate loci for altitude specific adaptation, we correlated allele frequencies with an environmental variable summarizing altitude and climate using Bayenv2.0. A total of 2978 SNPs showed the highest score possible for *Z* and were located in 616 genes.

At the SNP level, the overlap between the Bayenv2.0- and F_ST_-based approaches was low (3.02%, 90 SNPs). Of these, 42 SNPs were located in 39 genes (see Additional file 10). Genes containing top Bayenv2.0 SNPs matched 216 of the F_ST_-based top 5% differentiated genes.

### Functional inference of candidate genes

Gene set enrichment analysis of the 2944 genes containing top 0.5% SNPs indicated the presence of 62 enriched biological processes (see Additional file 11a). These involve many pathways, some of which are centrally important for stress response, cell growth, carbohydrate transport, and both asexual and sexual reproduction. For example among the significantly enriched categories we found biological processed like response to abiotic stimulus, growth, gene expression, RNA processing, translation, fungal-type cell wall polysaccharide biosynthetic process, catabolic processes, protein N-linked glycosylation, trehalose biosynthetic process, and developmental process involved in reproduction. GO enrichment of the 595 genes containing SNPs differentially fixed between the low (1 to 4) and high-altitude (6) genetic groups resulted in 38 enriched biological processes, including sexual and asexual reproduction, trehalose biosynthesis, growth, response to oxidative stresses, cell wall and ribosome biogenesis, and gene expression (see Additional file 11b).

GO enrichment of the top 1% Bayenv2.0 SNPs indicated that 23 biological processes were enriched (Table 3, see Additional file 12). Among the biological processes likely involved in adaptation to altitude we found localization, signal transduction, DNA repair, lipid modification, histone methylation, catabolic processes, and cell-wall biogenesis.

**Table 3:** GO enriched categories for top 1% *Z* Bayenv2.0 environmentally associated SNPs

Term ID	Top 1% Bayenv2.0 *Z*	Annotated	Significant	Top *Z*	Frequency	Log10 *P*
GO:0051179	localization	667	105	66	17.86%	−1.48
GO:0007154	cell communication	142	26	15	4.36%	−1.46
GO:0007165	signal transduction	126	23	12	3.80%	−1.33
GO:0090305	nucleic acid phosphodiester bond hydrolysis	17	7	5	2.51%	−2.48
GO:0006820	anion transport	60	14	9	2.20%	−1.77
GO:0006281	DNA repair	115	25	18	1.95%	−2.34
GO:0043414	macromolecule methylation	17	7	3	1.18%	−2.48
GO:0009066	aspartate family amino acid metabolic process	34	10	3	0.86%	−2.10
GO:0006553	lysine metabolic process	10	4	1	0.37%	−1.53
GO:0030258	lipid modification	16	6	3	0.25%	−1.96
GO:0016226	iron-sulfur cluster assembly	11	4	2	0.25%	−1.38
GO:0031163	metallo-sulfur cluster assembly	11	4	2	0.25%	−1.38
GO:0008213	protein alkylation	6	3	2	0.20%	−1.51
GO:0006479	protein methylation	6	3	2	0.20%	−1.51
GO:0046834	lipid phosphorylation	6	3	1	0.11%	−1.51
GO:0046854	phosphatidylinositol phosphorylation	6	3	1	0.11%	−1.51
GO:0006614	SRP-dependent cotranslational protein targeting to membrane	7	4	3	0.10%	−2.17
GO:0030259	lipid glycosylation	5	3	2	0.08%	−1.77
GO:0016571	histone methylation	5	3	2	0.03%	−1.77
GO:0042176	regulation of protein catabolic process	10	4	2	0.02%	−1.53
GO:0006273	lagging strand elongation	3	3	1	0.01%	−2.68
GO:0008608	attachment of spindle microtubules to kinetochore	4	3	3	0.01%	−2.12
GO:0070592	cell wall polysaccharide biosynthetic process	7	3	2	0.00%	−1.31
	Total unique	983	185	110		

### Ecophysiology

**Fig. 4:**
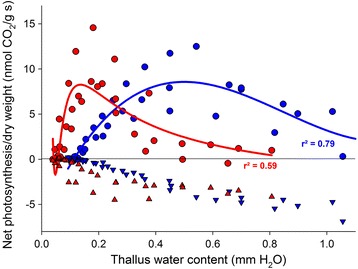
Photosynthetic CO_2_ gas exchange of *L. pustulata* highland (population 6; *blue*) and lowland population (populations 1 to 5; *red*) related to thallus water content (TWC). TWC is expressed as mm “precipitation”. Polynomic regressions lines are indicated with their r^2^ value. *Circles* = net CO_2_ uptake, *triangles* = dark respiration

Multi-locus genotyping grouped the samples into two genetic groups, one composed of all thalli from population 6 (G1), and one composed of thalli from all remaining sites (G2). Differentiated SNPs in all six markers were monomorphic between the two groups (see Additional file 3, Additional file 13). The genetic separation coincided with differences in anatomy and physiological responses to thallus water content (WC; Fig. 4) and high light conditions (see Additional file 14). First, G1 thalli had higher biomass (*p* = 0.002), and higher chlorophyll a + b content per surface area unit (*p* = 0.002) (Table 4). Second, G1 thalli needed higher WC for reaching maximal NP (0.5498 ± 0.104 mm H_2_O) compared to G2 thalli (0.175 ± 0.0 mm H_2_O; Fig. 4). In addition, G1 thalli reached their maximum NP rates (>90% of max value) at lower light intensity (see Additional file 14) (*p* = 0.003). In relation to surface area unit, G1 thalli fixed almost three times as much CO_2_. In relation to thallus dry weight G1 and G2 specimens did not significantly differ in their CO_2_ fixation rates (*p* = 0.65) (see Additional file 14, Additional file 15).

**Table 4:** Dry weight and chlorophyll a + b content of the different populations (*N* = 3 for each of the populations)

	Population 1	Population 2	Population 3	Population 4	Population 5	Population 6
Dry weight [mg/cm^2^]	25.99 ± 12.43	22.65 ± 3.10	23.59 ± 0.48	27.54 ± 7.63	30.75 ± 5.24	120.96 ± 58.71
Chlorophyll a + b [μg/cm^2^] surface area	38.28 ± 23.54	28.72 ± 10.77	19.67 ± 7.86	27.74 ± 6	22.9 ± 0.93	127.33 ± 40.0
Chlorophyll a + b [μg/mg] dry weight	1.42 ± 0.21	1.32 ± 0.612	0.83 ± 0.33	1.02 ± 0.16	0.63 ± 0.12	1.09 ± 0.18

## Discussion

Temperature and precipitation drive large-scale distribution patterns of lichens. It is thus expected that much of the signal of adaptation among lichen populations should occur along these gradients [60]. Here we presented the first genome-based analysis of population differentiation associated with an environmental gradient for a lichen-forming fungus. The studied populations underwent several generations of asexual reproduction, as only rarely sexual structures (apothecia) were observed in any of the sites. Thus, by applying Pool-seq resequencing to large population samples, we were able to track the frequencies of diverged long-lived lineages in each of the sites, and describe genome-wide population divergence in relation to changes in altitude.

Our study revealed significant differentiation and structure of *L. pustulata* populations. We found two genetic clusters along the gradient. One cluster is predominant at low elevations (up to ~800 m a.s.l.), while the other is predominant at high elevations (~1300 m a.s.l.). Our data suggest extensive admixture of these clusters at ~1100 m a.s.l. Given the high number of reciprocally fixed SNPs between the clusters and the high levels of clonal propagation in this fungal species, possible explanations for the observed pattern include ancient divergence, and a combination of limited gene flow, long generation times, and strong environmental filtering. Unfortunately there are no estimates for sexual or asexual generation times in *L. pustulata* from which we could calculate the age of the split between the clusters. Thus it is currently impossible to formally distinguish between the above scenarios. Ancient population splits and high genomic divergence have been reported in non-lichenized fungi. For example, strong genomic divergence, evidence for ancient population splits and introgression between subpopulations were inferred for the human pathogens *Coccidioides immitis* and *C. posadasii* [61]*.* Strong genetic structure was also found in locally adapted subpopulations of *Neurospora crassa* [5]. Numerous theoretical and empirical studies suggest that strong population divergence among continuously distributed populations may be caused by selection along environmental gradients promoting adaptation to different environmental conditions and ultimately impeding gene flow [62–64]. Interestingly, the genetic clusters of *L. pustulata* correspond to the major bioclimatic zones covered by our transect, with their admixture zone coinciding with the transition between the Mediterranean and the temperate-oceanic climate. Therefore, environmental filters likely contribute to the observed genetic structure.

To search for loci putatively involved in environmental adaptation, we detected allele-climate associations. Heat, drought, and intense light belong to the selective forces that cause differentiation among and within plant species in Mediterranean ecosystems [65]. We thus expected to find diversifying selection in those loci associated with pathways of the fungal environmental stress response (ESR). The ESR is in fact a common feature in the response of fungi to different environments, and it is responsible for initiating gene expression that protects the cell against stress [66, 67]. In yeasts, the ESR includes ~900 genes and requires a coordinated effort from multiple pathways, including signal transduction molecules, enzymes involved in cellwall biogenesis and maintenance, genes responsible for regulation of transcription, post-translational modification, and enzymes with proteolytic or antioxidant activities [68]. We found representative genes of each of the above pathways in our set of candidates for altitudinal adaptation. One example is alpha-ketoglutarate-dependent dioxygenase, a gene that is involved in the catalysis of taurine. Taurine is a solute required in osmoregulation, and has been linked to the survival of the fungus *Ochroconis mirabilis* in different habitats [69]. In the same functional category, we found candidate SNPs in genes such as a flavin-binding monooxygenase, and in the putative essential subunit of U3-containing 90S preribosome (*NOP9*), which has been reported among the 71 essential genes required for oxidative stress tolerance in *Saccharomyces cerevisiae* [70]. Additionally we found the calcium channel subunit *Cch1*, which has been reported to be involved in the Ca^2+^ release in response to exogenous oxidative stress in yeast [71], and a putative flap endonuclease, which is part of the base-excision repair pathway that removes lesions resulting from exposure to reactive oxygen species in yeast [72, 73]. Among the candidates involved in thermal stress response, we found the small heat shock protein *Hsp20*, glutamate carboxypeptidase, and a putative thermotolerance protein. Other candidates for diversifying selection include genes involved in the regulation of gene transcription, in particular *Isw1*, a gene that functions in parallel with the *NuA4* and *Swr1* complexes to regulate stress-induced gene transcription via chromatin remodeling in yeast [74]; interestingly, also *Swr1* was detected among the candidates. Furthermore we found genes putatively involved in cellwall integrity and filamentous growth pathways (septin [75]), and in UV-damage response (UV-damage endonuclease [76, 77]).

Several of the top differentiated genes containing environmentally associated SNPs were also involved in the ESR [78]. In particular we found genes linked to signal transduction and cell-wall integrity pathways, such as the transmembrane cellwall sensor *Wsc4*, a MAPKKK-cascade protein kinase, a two-component osmosensing histidine kinase, and a calcium/calmodulin-dependent kinase (*CAMK*). *CAMK* proteins were reported to be involved in thermotolerance and oxidative stress survival in *Neurospora crassa* [79]. The presence of a putative trehalose-phosphate synthase, and of a number of ribosomal proteins genes, RNA helicases, and thioredoxin among the candidates suggests that many genes involved in redox homeostasis and also important for cold-shock response are putative targets of selection [80]. It is therefore tempting to speculate that adaptation to different temperatures, in particular cold-shock, is a driver of population differentiation in *L. pustulata*, given a difference of ~6 °C in the mean annual temperature between top and bottom of the cline, and frequent winter frost above 1100 m a.s.l.

The overlap between environmentally associated SNPs and F_ST_-based outliers was low in our study. As in other studies of local adaptation [81–83], different approaches to identify candidate loci yielded different sets of candidates. This is probably due to the effects of population structure, and the parameters used for the environmental correlation. The limited overlap between sets of outliers indicates that selection along the gradient occurs mainly at the scale of local populations, and only partially at the evolutionary scale of the ancestral genetic groups [81]. Another reason for this difference may be attributed to the presence of environmental drivers that do not covary with chosen environmental factors, either biotic (e.g., interactions with photosynthetic partners, bacteria, or pathogens, and intra- and inter-specific competition), or abiotic (e.g., cloud cover, wind speed). In addition, covariance of population structure with the environment has been shown to make the method correcting for neutral population structure over-conservative [81]. Thus, candidates identified via methods that do not adjust for population structure should not be ignored, just treated carefully as their interpretation is necessarily *post hoc*. Future studies of *L. pustulata* will have to include more populations and replicate independent clines to fully disentangle demography from local selection.

Many candidate genes have known roles in stress response and growth regulation, and it is thus tempting to hypothesize that variation at these loci might affect fitness-related traits. Our ecophysiological experiments showed the presence of genetic lineages with differential fitness under different environmental conditions along the cline. The high altitude group seems to be better adapted to more humid conditions and to lower light intensities than the lowland group. In particular, samples from this population had thicker thalli and thus more biomass per area unit. In poikilohydric organisms such as lichens, an increased fungal biomass may be beneficial at wetter high-elevation sites and where winds speed up drying, especially on the exposed rocky outcrops where the species lives. This is because a higher fungal biomass may lead to i) higher mechanical stability against mechanical damage, ii) prolongation of the wet phase for an increase of the active period [84], and iii) acceptance of higher thallus water content [85]. The latter point is supported by our finding that high-altitude individuals need more water for maximal net photosynthesis than low-altitude ones. Moreover, under similar light and humidity conditions, low-altitude individuals would eventually die because of respiration rates exceeding carbon fixation rates. Structural changes towards improved thallus hydration in relation to improved photosynthetic exploitation were shown to drive acclimation in populations from different slopes in *Ramalina capitata* [86], between vagrant and attached morphs of *Cetraria aculeata* [21], between shaded and exposed populations in the Antarctic endemic *Catillaria corymbosa* [87], and between populations from different biomes in *Psora decipiens* [88]. Thallus thickness is a property controlled by the mycobiont and thus is related to the mycobiont’s response capability to environmental conditions [19]. Future genome-wide studies on the photobiont are required in order to elucidate the relative role of each symbiont in shaping the lichen’s response to the environment. Studies on the ecological benefits of phenotypic plasticity in lichens are still in their infancy, and the genomic basis of physiologically-relevant traits is far from being understood. Our results suggest that a surprisingly high, possibly adaptive, genetic diversity is responsible for anatomical and physiological differences between ecomorphs of a morphologically homogeneous lichen species. Further genomic research and physiological experiments based on replicate populations from different geographic areas, and comparisons with other lichen species will enable us to test the aforementioned hypothesis.

Adaptation to divergent environments promotes environmental specialization and reproductive isolation among fungal populations [5, 9]. In fact, the exclusion of immigrating individuals due to higher fitness of local genotypes (i.e., isolation by adaptation) can also lead to reproductive isolation resulting in little or no effective gene flow between geographically close populations [89]. This process, known as ecological speciation, interprets reproductive isolation as a by-product of adaptation to divergent environments. Our analysis of fixed variation between the highly differentiated genetic clusters of *L. pustulata* showed that several genes involved in sexual reproduction were significantly enriched, supporting the hypothesis of reproductive isolation. This is interesting, because we did not observe morphological evidence for sexual reproduction, such as presence of apothecia. Additional indication for reproductive isolation stems from the observation that genomic divergence between clusters is not limited to a few genomic areas, but rather widely dispersed across the genome. It involves many pathways centrally important for the response to environmental signals and stress, gene expression, growth, and metabolism. Overall, the high level of genomic divergence, and the presence of physiological differences between genetic clusters suggest the existence of *L. pustulata* ecotypes adapted to the Mediterranean and temperate-oceanic bioclimatic zone.

### Conclusions

Pool-seq genome resequencing is a cost-effective and powerful approach to assess allelic diversity in populations, and to identify genes that are potentially under selection [90–92]. However, this method also has limitations, mainly associated with the increased impact of sequencing errors and resampling of alleles. To circumvent these issues, we created equimolar genomic libraries based on high sampling density, sequenced each population at high coverage, and used strict thresholds for sequence quality filtering. Furthermore, we based the calculations of genetic diversity measures on subsampled data to avoid coverage bias, and used analysis tools specifically adapted to Pool-seq data. An intrinsic limitation of pooled sequencing is the loss of linkage disequilibrium information. The proposed candidates may not be under selection but may have been detected because of being linked to the actual targets of selection, or to other loci that diverged because of different evolutionary processes (e.g., genetic drift). This limitation can only be overcome by using different sequencing strategies, such as genome resequencing of individuals.

Our exploratory work into the genomics of adaptation of a lichen-forming fungus reveals numerous loci and pathways putatively involved in environmental adaptation, including many loci shown in other fungi to be linked to temperature and UV-radiation stress response. Such genes provide excellent targets for further investigations. Future studies based on individual genotyping, possibly including replicate populations from different regions, additional physiological analyses including more samples, and quantitative trait locus mapping experiments of the candidate genes in controlled and field settings will help to elucidate the drivers of local adaptation in this and other fungal species.

## Additional files

Additional file 1: Temperature data (in °C) collected between May 28th 2014 and June 2nd 2015 from loggers positioned at the level of *L. pustulata* thalli (two loggers per population). (PDF 113 kb)

Additional file 2: Plot of the first two axes of a PCA showing high correlation between 19 bioclimatic variables and altitude for the six populations along the cline. (PDF 524 kb)

Additional file 3: Genetic characteristics of the six loci used to genotype 18 *L. pustulata* samples for anatomical and physiological measurements. (PDF 113 kb)

Additional file 4: Distribution of nucleotide diversity, measured as Tajima's *D*, across 309 non-overlapping sliding windows of 10-kb. For the six scatterplots on the left, the data are plotted in ascending order of mean diversity across the six populations (solid line) together with the respective 95% confidence interval (grey envelope). Black crosses depict population-specific outlier SNPs. For the boxplot on the right, the data are summarized for each population across all windows. (PDF 517 kb)

Additional file 5: a) The mean and standard deviation for three standard population genetic parameters, *π*, Watterson's θ (θ_W_), and Tajima's *D* in non-overlapping 10-kb windows across the genome of *L. pustulata*. b) Pairwise Tukey contrasts based on linear mixed effect models for each measure and population. (PDF 446 kb)

Additional file 6: NJ trees of the correlation matrix obtained with Bayenv2.0 and the pairwise F_ST_ values. (PDF 165 kb)

Additional file 7: Average pairwise F_ST_ (lower triangle) and pairwise correlations of Bayenv2.0 correlation matrix among allele frequencies (upper triangle). (PDF 81 kb)

Additional file 8: Three-(a) and four-(b) population test of the *L. pustulata* populations. The test was performed on 722,401 high confidence SNPs. Bold values indicate significant *f3* (if z < 0) or *f4* statistics (if |z| < 3). (PDF 236 kb)

Additional file 9: Parameter estimates from MIGRATE-N based on 49 loci for pairs of three genetic groups of *L. pustulata* found along the altitudinal gradient (A: populations 1 to 4, B: population 5; C: population 6). (PDF 110 kb)

Additional file 10: Allele frequency and annotation for the 42 environmentally associated (top *Z* Bayenv2.0 score), highly differentiated (top 0.5% F_ST_ distribution) SNPs. (PDF 126 kb)

Additional file 11: GO enriched categories for a) top 0.5% highly differentiated SNPs, b) top 0.5% highly differentiated SNPs differentially fixed between populations 1–4 and population 6. (PDF 228 kb)

Additional file 12: List of significantly enriched GO terms for environmentally associated genes. Gene names in bold indicate genes containing top *Z* Bayenv2.0 SNPs. (PDF 103 kb)

Additional file 13: Neighbor-joining tree of the aligned concatenated gene fragments of the six loci at which we genotyped 18 thalli *L. pustulata* used for the anatomical and ecophysiological measurements. Samples in bold and underlined (*N* = 6) were used for the gas exchange experiments. (PDF 165 kb)

Additional file 14: Light (a), temperature (b) curves for 6 thalli representing two genetic groups (1: pop. 6, high altitude; 2: populations 1 to 5, low altitude). (PDF 556 kb)

Additional file 15: Net photosynthesis at optimal thallus water content and optimal temperature for genetic group A (pop. 6, high altitude) and B (populations 1 to 5, low altitude). (PDF 94 kb)
