# Supplementary material for: Adaptive differentiation coincides with local bioclimatic conditions along an elevational cline in populations of a lichen-forming fungus
Source: BMC Evol Biol. 2017 Mar 31;17:93. doi: 10.1186/s12862-017-0929-8 (PMC5374679; doi:10.1186/s12862-017-0929-8)
Supplement: Supplementary file 1 — Temperature data (in °C) collected between May 28th 2014 and June 2nd 2015 from loggers positioned at the level of L. pustulata thalli (two loggers per population). (PDF 113 kb) [file 12862_2017_929_MOESM1_ESM.pdf]

Additional file 1. Temperature data (in °C) collected between May 28th 2014 and June 2nd 2015 from loggers positioned at the level of *L. pustulata* thalli (two loggers per population).

**Months**	**Pool1**	**Pool2**	**Pool3**	**Pool4**	**Pool5**	**Pool6**
January	8.97	10.65	6.73	5.99	3.44	2.50
February	8.69	9.38	5.22	4.85	1.69	1.39
March	12.43	12.47	8.77	7.83	5.20	3.97
April	16.22	16.94	13.00	14.49	10.55	9.89
May	22.94	22.63	19.38	20.88	15.81	15.09
June	25.59	26.88	22.08	24.83	18.92	18.73
July	27.37	26.76	22.89	24.09	18.96	17.81
August	28.67	28.69	24.12	27.05	21.72	20.17
September	25.81	26.64	21.51	23.02	18.92	17.68
October	21.51	23.46	17.90	18.59	15.40	14.97
November	15.13	16.69	12.89	12.66	10.23	9.35
December	10.27	11.40	7.62	6.53	4.57	3.84
	18.63	19.38	15.18	15.90	12.12	11.28

**Indices**	**Pool1**	**Pool2**	**Pool3**	**Pool4**	**Pool5**	**Pool6**
average temperature coldest quarter (°C)	9.31	10.48	6.52	5.79	3.23	2.58
average temperature warmest quarter (°C)	27.21	27.44	23.03	25.32	19.87	18.90
max temperature of warmest month (°C)	53.28	49.80	42.59	53.55	42.59	40.35
min temperature of coldest month (°C)	0.79	1.31	-0.45	-2.06	-2.48	-3.73
average temperature coldest month (°C)	8.69	9.38	5.22	4.85	1.69	1.39
