# Supplementary material for: Adaptive differentiation coincides with local bioclimatic conditions along an elevational cline in populations of a lichen-forming fungus
Source: BMC Evol Biol. 2017 Mar 31;17:93. doi: 10.1186/s12862-017-0929-8 (PMC5374679; doi:10.1186/s12862-017-0929-8)

**Additional file 2**. Plot of the first two axes of a PCA showing high correlation between 19 bioclimatic variables and altitude for the six populations along the cline.

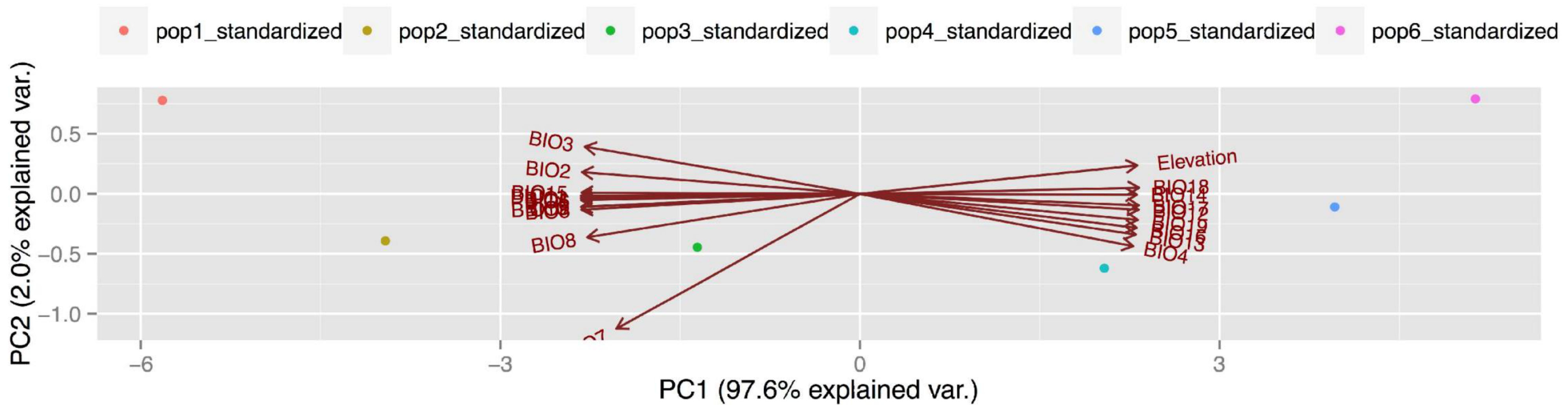

Supplement: Supplementary file 2 — Plot of the first two axes of a PCA showing high correlation between 19 bioclimatic variables and altitude for the six populations along the cline. (PDF 524 kb) [file 12862_2017_929_MOESM2_ESM.pdf]
