# Supplementary material for: Adaptive differentiation coincides with local bioclimatic conditions along an elevational cline in populations of a lichen-forming fungus
Source: BMC Evol Biol. 2017 Mar 31;17:93. doi: 10.1186/s12862-017-0929-8 (PMC5374679; doi:10.1186/s12862-017-0929-8)
Supplement: Supplementary file 3 — Genetic characteristics of the six loci used to genotype 18 L. pustulata samples for anatomical and physiological measurements. (PDF 113 kb) [file 12862_2017_929_MOESM3_ESM.pdf]

Additional file 3. Genetic characteristics of the six loci used to genotype 18 *L. pustulata* samples for anatomical and physiological measurements.

Gene	Primer	Primer sequence	Annotation	Position (bp)*	Allele pops. 1 to 5	Allele pop. 6
LPUS_05581	forward<br>reverse	CATCCAATTTGCGCGTAGTGTG<br>GAATCGCTGGTGGTCAGGTG	Hsp90-like protein	1775	C	T
LPUS_03073	forward<br>reverse	GAAGTGGCAAACCCTCGATCG<br>CTATAGTCGGTTTGGCAAATCTTAC	polyketide synthase	3081	A	C
LPUS_11054	forward<br>reverse	ATCAAGGACCGTCGGCCTTTTG<br>CAGTGACAGCACCAATTGGCAC	protein kinase C	1581	A	G
LPUS_02974	forward<br>reverse	CTTGGAAAGCATATGGCTATTG<br>CTAGAGACTCTACCAAACTGG	thermotolerance protein	683	T	G
LPUS_02744	forward<br>reverse	GATCTGATCACTTACCTCGAC<br>CATGAGTCGTACTGAATTCATAG	metallo-beta-lactamase family protein	389	T	C
LPUS_03358	forward<br>reverse	ATGCCCAACCGCCGATTC<br>CTTATTCACCTCGTTATGCACATGAC	FAD-linked sulfhydryl oxidase Alr protein	246	G	A

* Positions in loci LPUS_05581, LPUS_03073 and LPUS_11054 are 0.5% top F_{ST}-based SNPs and refer to coding sequence position. Positions in loci LPUS_02974, LPUS_02744, and LPUS_03358 are top *Z* Bayenv2.0 SNPs and refer to coding sequence position.
