# Supplementary material for: Adaptive differentiation coincides with local bioclimatic conditions along an elevational cline in populations of a lichen-forming fungus
Source: BMC Evol Biol. 2017 Mar 31;17:93. doi: 10.1186/s12862-017-0929-8 (PMC5374679; doi:10.1186/s12862-017-0929-8)

Additional file 4. Distribution of nucleotide diversity, measured as Tajima's *D*, across 309 non-overlapping sliding windows of 10-kb. For the six scatterplots on the left, the data are plotted in ascending order of mean diversity across the six populations (solid line) together with the respective 95% confidence interval (grey envelope). Black crosses depict population-specific outlier SNPs. For the boxplot on the right, the data are summarized for each population across all windows.

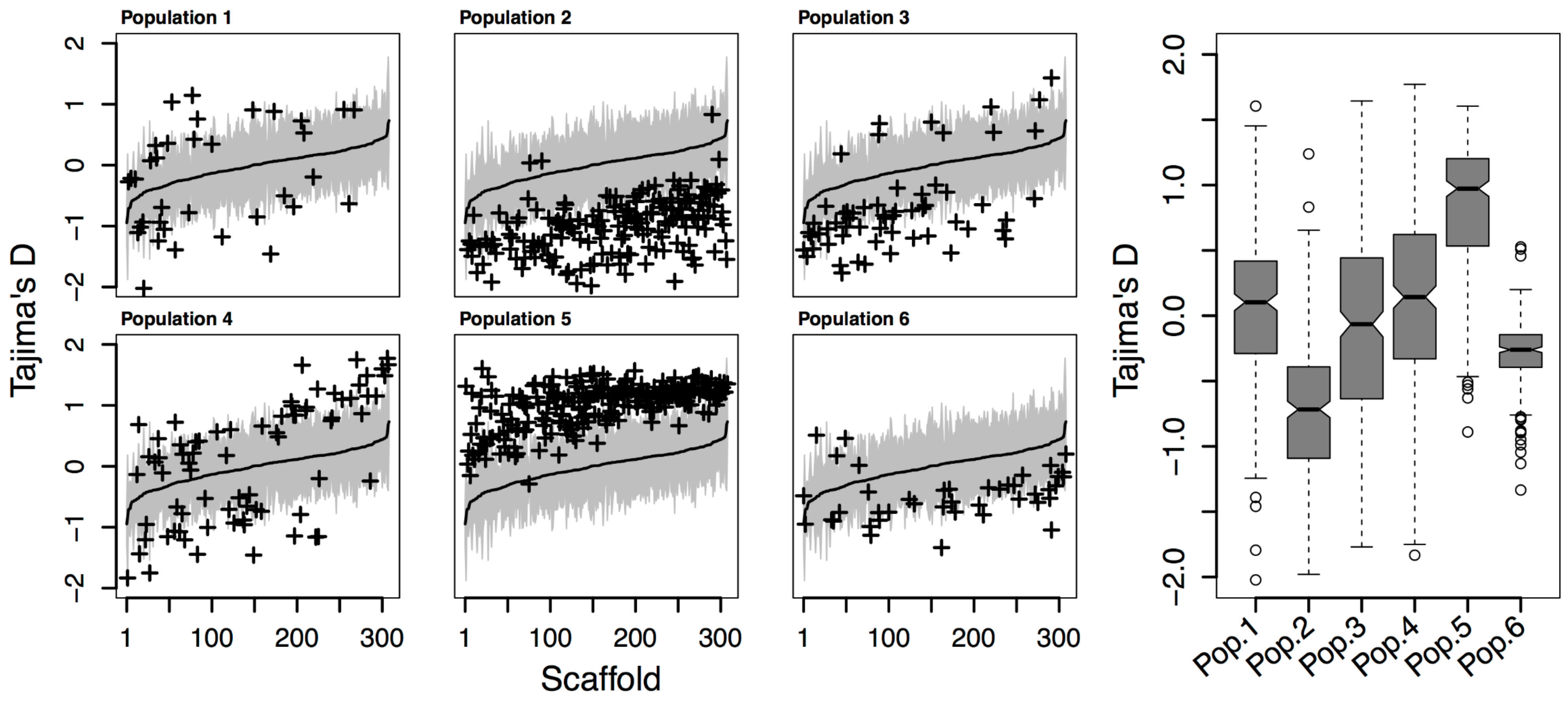

Supplement: Supplementary file 4 — Distribution of nucleotide diversity, measured as Tajima's D, across 309 non-overlapping sliding windows of 10-kb. For the six scatterplots on the left, the data are plotted in ascending order of mean diversity across the six populations (solid line) together with the respective 95% confidence interval (grey envelope). Black crosses depict population-specific outlier SNPs. For the boxplot on the right, the data are summarized for each population across all windows. (PDF 517 kb) [file 12862_2017_929_MOESM4_ESM.pdf]
