# Supplementary material for: Adaptive differentiation coincides with local bioclimatic conditions along an elevational cline in populations of a lichen-forming fungus
Source: BMC Evol Biol. 2017 Mar 31;17:93. doi: 10.1186/s12862-017-0929-8 (PMC5374679; doi:10.1186/s12862-017-0929-8)
Supplement: Supplementary file 5 — a) The mean and standard deviation for three standard population genetic parameters, π, Watterson's θ (θW), and Tajima's D in non-overlapping 10-kb windows across the genome of L. pustulata. b) Pairwise Tukey contrasts based on linear mixed effect models for each measure and population. (PDF 446 kb) [file 12862_2017_929_MOESM5_ESM.pdf]

Additional file 5. a) The mean and standard deviation for three standard population genetic parameters, π, Watterson's θ (θ_W), and Tajima's *D* in non-overlapping 10-kb windows across the genome of *L. pustulata*. b) Pairwise Tukey contrasts based on linear mixed effect models for each measure and population.

a)

π

	mean	sd
pop1	0.003751637	0.003492711
pop2	0.004406057	0.003354233
pop3	0.003681413	0.003369712
pop4	0.003592740	0.003333663
pop5	0.005505238	0.003858647
pop6	0.004120742	0.003494257

Tajima's D

	mean	sd
pop1	0.154169039	0.829874561
pop2	-0.839410129	0.712742622
pop3	0.049739133	0.925978082
pop4	0.198916837	0.967587202
pop5	1.111959530	0.539736077
pop6	-0.260107058	0.321729979

Watterson's θ

	mean	sd
pop1	0.003547405	0.003332516
pop2	0.005363743	0.003219011
pop3	0.003575289	0.003324172
pop4	0.003337400	0.003149108
pop5	0.004387760	0.003662431
pop6	0.004439896	0.003728312

b)

π

	Estimate	SE	z	P
pop2 - pop1	5.40E-04	9.80E-05	5.514	< 0.001 ***
pop3 - pop1	-1.70E-04	9.05E-05	-1.882	0.37178
pop4 - pop1	-3.17E-04	8.42E-05	-3.760	0.00195 **
pop5 - pop1	2.09E-03	1.71E-04	12.216	< 0.001 ***
pop6 - pop1	5.27E-04	1.74E-04	3.034	0.02381 *
pop3 - pop2	-7.10E-04	1.17E-04	-6.099	< 0.001 ***
pop4 - pop2	-8.57E-04	1.08E-04	-7.963	< 0.001 ***
pop5 - pop2	1.55E-03	1.91E-04	8.099	< 0.001 ***
pop6 - pop2	-1.34E-05	1.92E-04	-0.070	1
pop4 - pop3	-1.46E-04	9.47E-05	-1.544	0.59487
pop5 - pop3	2.26E-03	1.63E-04	13.830	< 0.001 ***
pop6 - pop3	6.97E-04	1.61E-04	4.320	< 0.001 ***
pop5 - pop4	2.40E-03	1.78E-04	13.469	< 0.001 ***
pop6 - pop4	8.43E-04	1.77E-04	4.760	< 0.001 ***
pop6 - pop5	-1.56E-03	8.16E-05	-19.102	< 0.001 ***

Watterson's θ

	Estimate	SE	z	P
pop2 - pop1	1.62E-03	8.53E-05	19.038	< 0.001 ***
pop3 - pop1	1.95E-05	7.38E-05	0.264	0.99978
pop4 - pop1	-3.62E-04	7.62E-05	-4.758	< 0.001 ***
pop5 - pop1	1.08E-03	1.21E-04	8.955	< 0.001 ***
pop6 - pop1	1.14E-03	1.32E-04	8.656	< 0.001 ***
pop3 - pop2	-1.60E-03	9.35E-05	-17.155	< 0.001 ***
pop4 - pop2	-1.99E-03	8.31E-05	-23.898	< 0.001 ***
pop5 - pop2	-5.42E-04	1.46E-04	-3.700	0.00252 **
pop6 - pop2	-4.84E-04	1.56E-04	-3.096	0.02013 *
pop4 - pop3	-3.82E-04	7.64E-05	-5.000	< 0.001 ***
pop5 - pop3	1.06E-03	1.16E-04	9.134	< 0.001 ***
pop6 - pop3	1.12E-03	1.27E-04	8.836	< 0.001 ***
pop5 - pop4	1.44E-03	1.33E-04	10.882	< 0.001 ***
pop6 - pop4	1.50E-03	1.43E-04	10.496	< 0.001 ***
pop6 - pop5	5.72E-05	7.29E-05	0.784	0.96445

Tajima's D

	Estimate	SE	z	P
pop2 - pop1	-0.88785	0.04516	-19.661	< 1e-04 ***
pop3 - pop1	-0.19458	0.04655	-4.180	0.00038 ***
pop4 - pop1	0.03933	0.04486	0.877	0.94734
pop5 - pop1	0.90731	0.03436	26.406	< 1e-04 ***
pop6 - pop1	-0.40573	0.03406	-11.911	< 1e-04 ***
pop3 - pop2	0.69326	0.05021	13.807	< 1e-04 ***
pop4 - pop2	0.92718	0.0524	17.695	< 1e-04 ***
pop5 - pop2	1.79516	0.04091	43.875	< 1e-04 ***
pop6 - pop2	0.48212	0.03551	13.576	< 1e-04 ***
pop4 - pop3	0.23391	0.04935	4.740	< 1e-04 ***
pop5 - pop3	1.10189	0.041	26.875	< 1e-04 ***
pop6 - pop3	-0.21114	0.04172	-5.061	< 1e-04 ***
pop5 - pop4	0.86798	0.04223	20.555	< 1e-04 ***
pop6 - pop4	-0.44506	0.04257	-10.455	< 1e-04 ***
pop6 - pop5	-1.31304	0.02206	-59.527	< 1e-04 ***

Signif.codes: 0 ‘***’ 0.001 ‘**’ 0.01 ‘*’ 0.05 ‘.’ 0.1 ‘ ’ 1

Note: Estimates for the mean pairwise difference deviate slightly from the difference between the real mean values. This is because the models incorporate a random structure that allows each population an independent slope across the scaffolds.
