# Supplementary material for: Adaptive differentiation coincides with local bioclimatic conditions along an elevational cline in populations of a lichen-forming fungus
Source: BMC Evol Biol. 2017 Mar 31;17:93. doi: 10.1186/s12862-017-0929-8 (PMC5374679; doi:10.1186/s12862-017-0929-8)

Additional file 6. NJ trees of the correlation matrix obtained with Bayenv2.0 and the pairwise F_{ST} values.

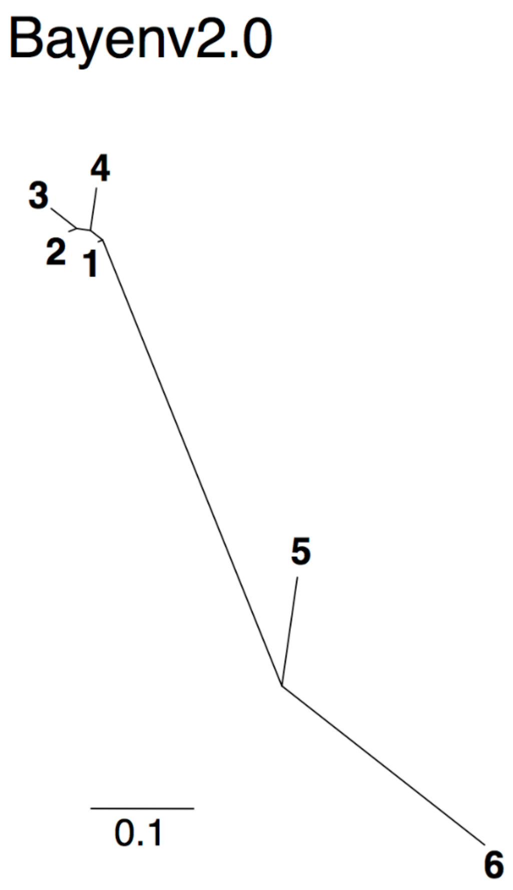

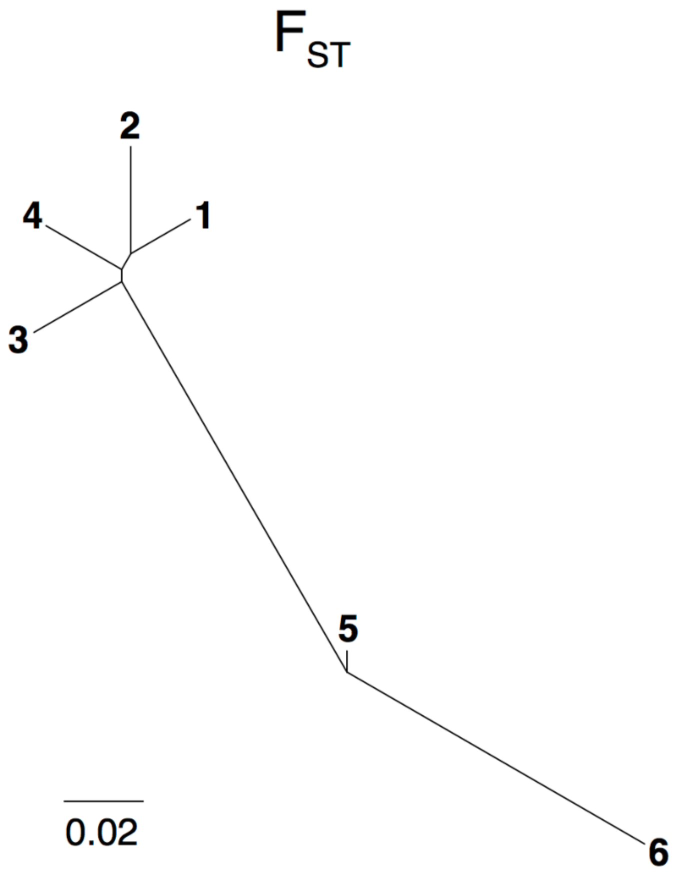

Supplement: Supplementary file 6 — NJ trees of the correlation matrix obtained with Bayenv2.0 and the pairwise FST values. (PDF 165 kb) [file 12862_2017_929_MOESM6_ESM.pdf]
