# Supplementary material for: Adaptive differentiation coincides with local bioclimatic conditions along an elevational cline in populations of a lichen-forming fungus
Source: BMC Evol Biol. 2017 Mar 31;17:93. doi: 10.1186/s12862-017-0929-8 (PMC5374679; doi:10.1186/s12862-017-0929-8)
Supplement: Supplementary file 7 — Average pairwise FST (lower triangle) and pairwise correlations of Bayenv2.0 correlation matrix among allele frequencies (upper triangle). (PDF 81 kb) [file 12862_2017_929_MOESM7_ESM.pdf]

Additional file 7. Average pairwise F_{ST} (lower triangle) and pairwise correlations of Bayenv2.0 correlation matrix among allele frequencies (upper triangle).

	Pool1	Pool2	Pool3	Pool4	Pool5	Pool6
Pool1	-	0.960	0.938	0.966	0.599	0.230
Pool2	0.045	-	0.910	0.950	0.572	0.209
Pool3	0.051	0.064	-	0.942	0.578	0.225
Pool4	0.044	0.055	0.050	-	0.639	0.278
Pool5	0.136	0.139	0.139	0.134	-	0.854
Pool6	0.230	0.236	0.225	0.231	0.082	-
