# Supplementary material for: Adaptive differentiation coincides with local bioclimatic conditions along an elevational cline in populations of a lichen-forming fungus
Source: BMC Evol Biol. 2017 Mar 31;17:93. doi: 10.1186/s12862-017-0929-8 (PMC5374679; doi:10.1186/s12862-017-0929-8)
Supplement: Supplementary file 8 — Three-(a) and four-(b) population test of the L. pustulata populations. The test was performed on 722,401 high confidence SNPs. Bold values indicate significant f3 (if z < 0) or f4 statistics (if |z| < 3). (PDF 236 kb) [file 12862_2017_929_MOESM8_ESM.pdf]

Additional file 8. Three-(a) and four-(b) population test of the *L. pustulata* populations. The test was performed on 722,401 high confidence SNPs. Bold values indicate significant *f3* (if z < 0) or *f4* statistics (if |z| < 3).

a) threepop

Population pairs	*f3* statistic	Std. error	*z*-score
pop2-pop1,pop3	0.0173775	0.000636731	27.2918
pop3-pop1,pop2	0.0219619	0.000666119	32.9699
pop1-pop2,pop4	0.0077401	0.000438286	17.6599
pop2-pop1,pop4	0.0162849	0.000581215	28.0187
pop4-pop1,pop2	0.016254	0.000594484	27.3413
pop1-pop2,pop5	0.0106111	0.00051858	20.4618
pop2-pop1,pop5	0.0134139	0.000617673	21.7169
pop5-pop1,pop2	0.086129	0.00145668	59.127
pop2-pop1,pop6	0.0151955	0.000664185	22.8784
pop6-pop1,pop2	0.180839	0.00255812	70.6922
pop1-pop3,pop4	0.0120108	0.000491765	24.4238
pop3-pop1,pop4	0.0165987	0.000586755	28.2889
pop4-pop1,pop3	0.0119833	0.000471311	25.4255
pop1-pop3,pop5	0.0129564	0.00056417	22.9654
pop3-pop1,pop5	0.015653	0.000552825	28.3146
pop5-pop1,pop3	0.0837838	0.00142035	58.9882
pop1-pop3,pop6	0.0156229	0.00069885	22.3551
pop3-pop1,pop6	0.0129865	0.00059892	21.6832
pop6-pop1,pop3	0.174046	0.00251771	69.1286
pop1-pop4,pop5	0.0124765	0.000497308	25.088
pop4-pop1,pop5	0.0115176	0.000469425	24.5356
pop5-pop1,pop4	0.0842637	0.00141382	59.5999
pop1-pop4,pop6	0.0111959	0.000573403	19.5254
pop4-pop1,pop6	0.0127982	0.000547682	23.3679
pop6-pop1,pop4	0.178472	0.00254889	70.0196
pop1-pop5,pop6	0.118825	0.00188847	62.921
**pop5-pop1,pop6**	**-0.0220845**	**0.000641762**	**-34.4123**
pop6-pop1,pop5	0.0708437	0.000983307	72.0464
pop2-pop3,pop4	0.0216482	0.000816396	26.5167
pop3-pop2,pop4	0.0176912	0.000650505	27.1962
pop4-pop2,pop3	0.0108907	0.000511801	21.2792
pop2-pop3,pop5	0.0197228	0.000841837	23.4282
pop3-pop2,pop5	0.0196166	0.000692171	28.3407
pop5-pop2,pop3	0.0798202	0.00138885	57.4723
pop2-pop3,pop6	0.0241708	0.000919722	26.2806
pop3-pop2,pop6	0.0151686	0.000765618	19.8122
pop6-pop2,pop3	0.171863	0.00248627	69.1251
pop2-pop4,pop5	0.0181503	0.00074528	24.3536
pop4-pop2,pop5	0.0143886	0.000614642	23.4097
pop5-pop2,pop4	0.0813927	0.00139318	58.4222
pop2-pop4,pop6	0.0186513	0.000820023	22.7448
pop4-pop2,pop6	0.0138876	0.000725204	19.15

Population pairs	*f3* statistic	Std. error	*z*-score
pop6-pop2,pop4	0.177383	0.00251119	70.637
pop2-pop5,pop6	0.123409	0.00194466	63.4606
**pop5-pop2,pop6**	**-0.0238661**	**0.000645081**	**-36.997**
pop6-pop2,pop5	0.0726253	0.000988204	73.4922
pop3-pop4,pop5	0.0161187	0.000578704	27.8532
pop4-pop3,pop5	0.0124632	0.000572428	21.7726
pop5-pop3,pop4	0.0833181	0.00139937	59.5395
pop3-pop4,pop6	0.0121717	0.000612925	19.8584
pop4-pop3,pop6	0.0164103	0.000707956	23.1798
pop6-pop3,pop4	0.17486	0.00251857	69.4286
pop3-pop5,pop6	0.118855	0.00185244	64.1613
**pop5-pop3,pop6**	**-0.019418**	**0.000651205**	**-29.8186**
pop6-pop3,pop5	0.0681772	0.000961074	70.9386
**pop5-pop4,pop6**	**-0.0233651**	**0.000653936**	**-35.7299**
pop6-pop4,pop5	0.0721243	0.00097807	73.7415

b) fourpop

Population pairs	*f4* statistic	Std. error	*z*-score
**(pop1-pop2) vs. (pop3-pop4)**	**0.00109258**	**0.000412011**	**2.65183**
(pop1-pop3) vs. (pop2-pop4)	0.00536324	0.000531731	10.0864
(pop1-pop4) vs. (pop2-pop3)	0.00427065	0.000554483	7.70205
(pop1-pop2) vs. (pop3-pop5)	0.00396359	0.000547318	7.24184
(pop1-pop3) vs. (pop2-pop5)	0.00630886	0.000624146	10.108
(pop1-pop5) vs. (pop2-pop3)	0.00234527	0.000701747	3.34204
(pop1-pop2) vs. (pop3-pop6)	0.00218204	0.000655148	3.33061
(pop1-pop3) vs. (pop2-pop6)	0.00897538	0.00076377	11.7514
(pop1-pop6) vs. (pop2-pop3)	0.00679333	0.000845915	8.03076
(pop1-pop2) vs. (pop4-pop5)	0.00287101	0.000477792	6.00891
(pop1-pop4) vs. (pop2-pop5)	0.00473637	0.000511838	9.25365
(pop1-pop5) vs. (pop2-pop4)	0.00186536	0.000563857	3.30821
**(pop1-pop2) vs. (pop4-pop6)**	**0.00108946**	**0.00060498**	**1.80081**
(pop1-pop4) vs. (pop2-pop6)	0.00345581	0.000620346	5.57078
(pop1-pop6) vs. (pop2-pop4)	0.00236635	0.000691664	3.42125
(pop1-pop2) vs. (pop5-pop6)	-0.00178155	0.000354755	-5.02193
(pop1-pop5) vs. (pop2-pop6)	0.108214	0.00184589	58.6242
(pop1-pop6) vs. (pop2-pop5)	0.109995	0.00181925	60.4619
**(pop1-pop3) vs. (pop4-pop5)**	**0.00094562**	**0.000566741**	**1.66852**
**(pop1-pop4) vs. (pop3-pop5)**	**0.000465714**	**0.000496**	**0.938938**
**(pop1-pop5) vs. (pop3-pop4)**	**-0.000479907**	**0.000548285**	**-0.875287**
(pop1-pop3) vs. (pop4-pop6)	0.00361214	0.000714493	5.05553
**(pop1-pop4) vs. (pop3-pop6)**	**-0.000814842**	**0.000583135**	**-1.39735**
(pop1-pop6) vs. (pop3-pop4)	-0.00442698	0.000688982	-6.42539
(pop1-pop3) vs. (pop5-pop6)	0.00266652	0.0003691	7.22437
(pop1-pop5) vs. (pop3-pop6)	0.105868	0.00179605	58.9451

**Population pairs**	***f4* statistic**	**Std. error**	***z*-score**
(pop1-pop6) vs. (pop3-pop5)	0.103202	0.00180108	57.2999
(pop1-pop4) vs. (pop5-pop6)	-0.00128056	0.000357602	-3.58096
(pop1-pop5) vs. (pop4-pop6)	0.106348	0.00180608	58.8834
(pop1-pop6) vs. (pop4-pop5)	0.107629	0.00181726	59.2259
**(pop2-pop3) vs. (pop4-pop5)**	**-0.00192539**	**0.000708844**	**-2.71624**
(pop2-pop4) vs. (pop3-pop5)	-0.00349788	0.000617453	-5.66502
**(pop2-pop5) vs. (pop3-pop4)**	**-0.00157249**	**0.00058333**	**-2.69571**
**(pop2-pop3) vs. (pop4-pop6)**	**0.00252268**	**0.000868248**	**2.90548**
(pop2-pop4) vs. (pop3-pop6)	-0.00299688	0.000741656	-4.0408
(pop2-pop6) vs. (pop3-pop4)	-0.00551956	0.000713615	-7.73465
(pop2-pop3) vs. (pop5-pop6)	0.00444807	0.000437333	10.1709
(pop2-pop5) vs. (pop3-pop6)	0.103686	0.00176235	58.834
(pop2-pop6) vs. (pop3-pop5)	0.0992382	0.00178283	55.6632
**(pop2-pop4) vs. (pop5-pop6)**	**0.000500995**	**0.000424606**	**1.17991**
(pop2-pop5) vs. (pop4-pop6)	0.105259	0.00177071	59.4445
(pop2-pop6) vs. (pop4-pop5)	0.104758	0.00180329	58.0924
(pop3-pop4) vs. (pop5-pop6)	-0.00394707	0.000361426	-10.9208
(pop3-pop5) vs. (pop4-pop6)	0.102736	0.00179269	57.3084
(pop3-pop6) vs. (pop4-pop5)	0.106683	0.00178986	59.6043
