# Supplementary material for: Adaptive differentiation coincides with local bioclimatic conditions along an elevational cline in populations of a lichen-forming fungus
Source: BMC Evol Biol. 2017 Mar 31;17:93. doi: 10.1186/s12862-017-0929-8 (PMC5374679; doi:10.1186/s12862-017-0929-8)
Supplement: Supplementary file 9 — Parameter estimates from MIGRATE-N based on 49 loci for pairs of three genetic groups of L. pustulata found along the altitudinal gradient (A: populations 1 to 4, B: population 5; C: population 6). (PDF 110 kb) [file 12862_2017_929_MOESM9_ESM.pdf]

Additional file 9. Parameter estimates from MIGRATE-N based on 49 loci for pairs of three genetic groups of *L. pustulata* found along the altitudinal gradient (A: populations 1 to 4, B: population 5; C: population 6).

Parameter	2.5%	25%	75%	97.5%	Median	Mean
Theta A	0.059	0.060	0.063	0.066	0.061	0.054
Theta B	0.060	0.063	0.066	0.068	0.065	0.065
Theta C	0.047	0.052	0.056	0.058	0.054	0.057
Theta 1*M B->A	0.000	0.600	10.200	19.800	9.300	5.570
Theta 2*M C->A	0.000	0.000	7.800	16.800	8.100	2.269
Theta 3*M A->B	351.000	384.600	411.600	439.200	397.500	395.394
Theta 4*M C>B	234.000	249.000	266.400	289.200	260.700	293.705
Theta 5*M A>C	0.000	1.200	12.000	22.200	10.500	6.756
Theta 6*M B>C	0.000	3.600	15.600	24.600	12.300	9.927
