# Supplementary material for: Adaptive differentiation coincides with local bioclimatic conditions along an elevational cline in populations of a lichen-forming fungus
Source: BMC Evol Biol. 2017 Mar 31;17:93. doi: 10.1186/s12862-017-0929-8 (PMC5374679; doi:10.1186/s12862-017-0929-8)
Supplement: Supplementary file 10 — Allele frequency and annotation for the 42 environmentally associated (top Z Bayenv2.0 score), highly differentiated (top 0.5% FST distribution) SNPs. (PDF 126 kb) [file 12862_2017_929_MOESM10_ESM.pdf]

Additional file 10. Allele frequency and annotation for the 42 environmentally associated (top *Z* Bayenv2.0 score) and highly differentiated (top 0.5% F_{ST} distribution) SNPs.

Gene name	SNP position	Reference allele	Pool 1	Pool 2	Pool 3	Pool 4	Pool 5	Pool 6	Annotation
LPUS_06124	1376	C	0:21:9:0:0:0	0:3:27:0:0:0	0:26:4:0:0:0	0:0:30:0:0:0	0:19:11:0:0:0	0:30:0:0:0:0	rRNA processing protein Nop9
LPUS_07657	3527	G	16:0:0:14:0:0	30:0:0:0:0:0	3:0:0:27:0:0	0:0:0:30:0:0	0:0:0:30:0:0	0:0:0:30:0:0	glutamate carboxypeptidase II
LPUS_06827	948	G	24:0:0:6:0:0	4:0:0:26:0:0	16:0:0:14:0:0	0:0:0:30:0:0	17:0:0:13:0:0	30:0:0:0:0:0	flap endonuclease 1
LPUS_04288	761	G	20:0:0:10:0:0	16:0:0:14:0:0	20:0:0:10:0:0	0:0:0:30:0:0	21:0:0:9:0:0	30:0:0:0:0:0	
LPUS_07610	3404	A	30:0:0:0:0:0	22:0:0:8:0:0	10:0:0:20:0:0	12:0:0:18:0:0	9:0:0:21:0:0	0:0:0:30:0:0	
LPUS_06021	2351	A	12:0:0:18:0:0	12:0:0:18:0:0	13:0:0:17:0:0	30:0:0:0:0:0	13:0:0:17:0:0	0:0:0:30:0:0	vacuolar protein sorting protein
LPUS_03230	109	A	30:0:0:0:0:0	2:0:0:28:0:0	19:0:0:11:0:0	1:0:0:29:0:0	0:0:0:30:0:0	0:0:0:30:0:0	reverse transcriptase domain protein
LPUS_05016	142	A	16:0:0:14:0:0	0:0:0:30:0:0	27:0:0:3:0:0	30:0:0:0:0:0	30:0:0:0:0:0	25:0:0:5:0:0	Wsc-domain containing protein
LPUS_06496	4656	T	0:9:21:0:0:0	0:0:30:0:0:0	0:17:13:0:0:0	0:30:0:0:0:0	0:13:17:0:0:0	0:0:30:0:0:0	helicase swr1
LPUS_04302	417	A	20:0:10:0:0:0	17:0:13:0:0:0	6:0:24:0:0:0	0:0:30:0:0:0	15:0:15:0:0:0	30:0:0:0:0:0	UV damage endonuclease
LPUS_04302	442	A	20:10:0:0:0:0	14:16:0:0:0:0	3:27:0:0:0:0	0:30:0:0:0:0	18:12:0:0:0:0	30:0:0:0:0:0	
LPUS_03358	246	G	0:0:0:30:0:0	12:0:0:18:0:0	0:0:0:30:0:0	13:0:0:17:0:0	7:0:0:23:0:0	30:0:0:0:0:0	fad-linked sulfhydryl oxidase Alr protein
LPUS_10824	710	G	0:0:0:30:0:0	3:0:0:27:0:0	2:0:0:28:0:0	23:0:0:7:0:0	17:0:0:13:0:0	30:0:0:0:0:0	
LPUS_09622	443	G	0:0:0:30:0:0	0:0:14:16:0:0	0:0:18:12:0:0	0:0:20:10:0:0	0:0:17:13:0:0	0:0:30:0:0:0	protein kinase
LPUS_09622	902	C	0:0:30:0:0:0	17:0:13:0:0:0	19:0:11:0:0:0	25:0:5:0:0:0	16:0:14:0:0:0	30:0:0:0:0:0	
LPUS_09622	1200	A	30:0:0:0:0:0	10:0:20:0:0:0	12:0:18:0:0:0	6:0:24:0:0:0	11:0:19:0:0:0	0:0:30:0:0:0	
LPUS_09622	1290	A	30:0:0:0:0:0	7:0:0:23:0:0	10:0:0:20:0:0	8:0:0:22:0:0	13:0:0:17:0:0	0:0:0:30:0:0	
LPUS_02236	4121	C	0:12:18:0:0:0	0:14:16:0:0:0	0:20:10:0:0:0	0:30:0:0:0:0	0:11:19:0:0:0	0:0:30:0:0:0	90S preribosome SSU processome component Krr1
LPUS_02970	980	A	30:0:0:0:0:0	16:0:14:0:0:0	22:0:8:0:0:0	15:0:15:0:0:0	9:0:21:0:0:0	0:0:30:0:0:0	
LPUS_02974	683	T	0:30:0:0:0:0	1:19:0:10:0:0	1:24:0:5:0:0	0:20:0:10:0:0	0:9:0:21:0:0	0:0:0:30:0:0	thermotolerance protein
LPUS_01916	2259	C	1:0:18:11:0:0	0:0:26:4:0:0	9:0:15:6:0:0	0:0:30:0:0:0	0:0:12:18:0:0	0:0:0:30:0:0	septin
LPUS_07111	1299	A	5:0:0:25:0:0	16:0:0:14:0:0	12:0:0:18:0:0	30:0:0:0:0:0	10:0:0:20:0:0	0:0:0:30:0:0	
LPUS_05969	250	A	30:0:0:0:0:0	27:0:0:3:0:0	29:0:0:1:0:0	17:0:0:13:0:0	16:0:0:14:0:0	0:0:0:30:0:0	
LPUS_04289	839	A	15:0:0:15:0:0	10:0:0:20:0:0	12:0:0:18:0:0	30:0:0:0:0:0	12:0:0:18:0:0	0:0:0:30:0:0	Hsp20_like chaperone
LPUS_02732	179	T	0:7:23:0:0:0	0:18:12:0:0:0	0:17:13:0:0:0	0:30:0:0:0:0	0:13:17:0:0:0	0:0:30:0:0:0	phosphopantothenoylcysteine decarboxylase protein
LPUS_04375	216	T	0:27:3:0:0:0	0:30:0:0:0:0	0:4:26:0:0:0	0:3:27:0:0:0	0:0:30:0:0:0	0:0:30:0:0:0	flavin-binding monooxygenase
LPUS_07509	2843	G	0:0:0:30:0:0	4:0:0:26:0:0	22:0:0:8:0:0	10:0:0:20:0:0	23:0:0:7:0:0	30:0:0:0:0:0	calcium channel subunit Cch1
