# Supplementary material for: Adaptive differentiation coincides with local bioclimatic conditions along an elevational cline in populations of a lichen-forming fungus
Source: BMC Evol Biol. 2017 Mar 31;17:93. doi: 10.1186/s12862-017-0929-8 (PMC5374679; doi:10.1186/s12862-017-0929-8)
Supplement: Supplementary file 11 — GO enriched categories for a) top 0.5% highly differentiated SNPs, b) top 0.5% highly differentiated SNPs differentially fixed between populations 1–4 and population 6. (PDF 228 kb) [file 12862_2017_929_MOESM11_ESM.pdf]

**Additional file 11**. GO enriched categories for a) top 0.5% highly differentiated SNPs, b) top 0.5% highly differentiated SNPs differentially fixed between populations 1-4 and population 6.

a)

| term_ID | description | frequency | log10 p-value |
|---|---|---|---|
| GO:0051179 | localization | 17.86% | -1.4177 |
| GO:0010467 | gene expression | 16.70% | -2.2774 |
| GO:0016070 | RNA metabolic process | 13.71% | -1.5186 |
| GO:0019538 | protein metabolic process | 12.33% | -1.511 |
| GO:0007154 | cell communication | 4.36% | -1.6099 |
| GO:0006812 | cation transport | 3.98% | -3.3979 |
| GO:0023052 | signaling | 3.84% | -1.5547 |
| GO:0006508 | proteolysis | 3.71% | -2.2269 |
| GO:0006396 | RNA processing | 2.78% | -1.5196 |
| GO:0008610 | lipid biosynthetic process | 2.21% | -2.7852 |
| GO:0006310 | DNA recombination | 1.84% | -1.7484 |
| GO:0009057 | macromolecule catabolic process | 1.64% | -1.5659 |
| GO:0051641 | cellular localization | 1.64% | -1.4074 |
| GO:0008643 | carbohydrate transport | 1.47% | -3.8861 |
| GO:0051301 | cell division | 1.36% | -1.6692 |
| GO:0048519 | negative regulation of biological process | 1.24% | -2.062 |
| GO:0006022 | aminoglycan metabolic process | 1.10% | -1.769 |
| GO:0042254 | ribosome biogenesis | 1.03% | -1.9531 |
| GO:0016072 | rRNA metabolic process | 0.76% | -1.4634 |
| GO:0006023 | aminoglycan biosynthetic process | 0.73% | -1.4852 |
| GO:0022402 | cell cycle process | 0.53% | -2.1463 |
| GO:0009311 | oligosaccharide metabolic process | 0.51% | -1.4671 |
| GO:0016071 | mRNA metabolic process | 0.49% | -1.6899 |
| GO:0006928 | cellular component movement | 0.49% | -1.4689 |
| GO:0007059 | chromosome segregation | 0.37% | -1.6189 |
| GO:0009894 | regulation of catabolic process | 0.28% | -1.7404 |
| GO:0006413 | translational initiation | 0.27% | -1.4244 |
| GO:0031329 | regulation of cellular catabolic process | 0.26% | -1.4595 |
| GO:1901701 | cellular response to oxygen-containing compound | 0.23% | -2.8794 |
| GO:0000003 | reproduction | 0.21% | -2.7799 |
| GO:0007017 | microtubule-based process | 0.17% | -1.8159 |
| GO:0052646 | alditol phosphate metabolic process | 0.15% | -1.3021 |
| GO:0006072 | glycerol-3-phosphate metabolic process | 0.14% | -1.3021 |
| GO:0000272 | polysaccharide catabolic process | 0.14% | -2.3063 |
| GO:0019954 | asexual reproduction | 0.14% | -1.8404 |
| GO:0006298 | mismatch repair | 0.14% | -3.0862 |
| GO:1901071 | glucosamine-containing compound metabolic process | 0.13% | -2.0878 |
| GO:0016567 | protein ubiquitination | 0.11% | -1.5885 |
| GO:0010876 | lipid localization | 0.10% | -1.4618 |
| GO:0040007 | growth | 0.08% | -2.0357 |
| GO:0003006 | developmental process involved in reproduction | 0.08% | -2.2874 |
| GO:0000280 | nuclear division | 0.06% | -1.7701 |

| term_ID | description | frequency | log10 p-value |
|---|---|---|---|
| GO:0006816 | calcium ion transport | 0.05% | -1.5516 |
| GO:0005992 | trehalose biosynthetic process | 0.05% | -2.1707 |
| GO:0010565 | regulation of cellular ketone metabolic process | 0.04% | -1.4852 |
| GO:0051235 | maintenance of location | 0.03% | -2.1643 |
| GO:0006888 | ER to Golgi vesicle-mediated transport | 0.02% | -2.0878 |
| GO:0015914 | phospholipid transport | 0.02% | -1.7364 |
| GO:0032507 | maintenance of protein location in cell | 0.02% | -1.8196 |
| GO:0006271 | DNA strand elongation involved in DNA replication | 0.01% | -1.4852 |
| GO:0022616 | DNA strand elongation | 0.01% | -1.4852 |
| GO:0006487 | protein N-linked glycosylation | 0.01% | -2.3063 |
| GO:0018022 | peptidyl-lysine methylation | 0.00% | -1.3021 |
| GO:0030433 | ER-associated ubiquitin-dependent protein catabolic process | 0.00% | -1.7364 |
| GO:0030447 | filamentous growth | 0.00% | -1.5596 |
| GO:0042762 | regulation of sulfur metabolic process | 0.00% | -1.9248 |
| GO:0034248 | regulation of cellular amide metabolic process | 0.00% | -1.5516 |
| GO:0042318 | penicillin biosynthetic process | 0.00% | -2.1707 |
| GO:1900549 | N';N'';N'''-triacetylfusarinine C metabolic process | 0.00% | -1.7364 |
| GO:1900551 | N';N'';N'''-triacetylfusarinine C biosynthetic process | 0.00% | -1.7364 |
| GO:0051278 | fungal-type cell wall polysaccharide biosynthetic process | 0.00% | -2.1707 |
| GO:0036184 | asperthecin biosynthetic process | 0.00% | -1.3021 |

b)

| term_ID | description | frequency | log10 p-value |
|---|---|---|---|
| GO:0010467 | gene expression | 16.70% | -1.6542 |
| GO:0019538 | protein metabolic process | 12.33% | -1.7167 |
| GO:0031326 | regulation of cellular biosynthetic process | 9.00% | -2.4962 |
| GO:0044248 | cellular catabolic process | 7.95% | -1.3343 |
| GO:0006412 | translation | 4.70% | -1.3155 |
| GO:0006396 | RNA processing | 2.78% | -2.0969 |
| GO:0008643 | carbohydrate transport | 1.47% | -3.8539 |
| GO:0005976 | polysaccharide metabolic process | 1.08% | -1.9169 |
| GO:0042254 | ribosome biogenesis | 1.03% | -2.1226 |
| GO:0016071 | mRNA metabolic process | 0.49% | -1.7849 |
| GO:0006928 | cellular component movement | 0.49% | -1.6946 |
| GO:0052803 | imidazole-containing compound metabolic process | 0.46% | -1.5038 |
| GO:0006547 | histidine metabolic process | 0.45% | -1.5038 |
| GO:0090502 | RNA phosphodiester bond hydrolysis; endonucleolytic | 0.42% | -1.4405 |
| GO:0006397 | mRNA processing | 0.37% | -1.547 |
| GO:0009628 | response to abiotic stimulus | 0.31% | -1.5632 |
| GO:1901700 | response to oxygen-containing compound | 0.31% | -1.5117 |
| GO:0000272 | polysaccharide catabolic process | 0.14% | -1.7897 |
| GO:0019954 | asexual reproduction | 0.14% | -2.0921 |
| GO:0006298 | mismatch repair | 0.14% | -2.7852 |
| GO:0006112 | energy reserve metabolic process | 0.13% | -1.326 |
| GO:0048646 | anatomical structure formation involved in morphogenesis | 0.13% | -2.4609 |
| GO:0022414 | reproductive process | 0.12% | -1.4034 |

| term_ID | description | frequency | log10 p-value |
|---|---|---|---|
| GO:0051056 | regulation of small GTPase mediated signal transduction | 0.11% | -1.534 |
| GO:0016567 | protein ubiquitination | 0.11% | -2.0737 |
| GO:0046854 | phosphatidylinositol phosphorylation | 0.11% | -2.585 |
| GO:0040007 | growth | 0.08% | -1.6003 |
| GO:1903047 | mitotic cell cycle process | 0.08% | -2.9957 |
| GO:0044703 | multi-organism reproductive process | 0.07% | -1.7724 |
| GO:0005992 | trehalose biosynthetic process | 0.05% | -3.2757 |
| GO:0016049 | cell growth | 0.04% | -1.6112 |
| GO:0006888 | ER to Golgi vesicle-mediated transport | 0.02% | -2.9245 |
| GO:0006487 | protein N-linked glycosylation | 0.01% | -2.6655 |
| GO:0030010 | establishment of cell polarity | 0.01% | -2.15 |
| GO:0051784 | negative regulation of nuclear division | 0.01% | -2.1284 |
| GO:0071852 | fungal-type cell wall organization or biogenesis | 0.00% | -1.534 |
| GO:0033244 | regulation of penicillin metabolic process | 0.00% | -2.6198 |
| GO:0045460 | sterigmatocystin metabolic process | 0.00% | -1.326 |
