# Supplementary material for: Adaptive differentiation coincides with local bioclimatic conditions along an elevational cline in populations of a lichen-forming fungus
Source: BMC Evol Biol. 2017 Mar 31;17:93. doi: 10.1186/s12862-017-0929-8 (PMC5374679; doi:10.1186/s12862-017-0929-8)
Supplement: Supplementary file 12 — List of significantly enriched GO terms for environmentally associated genes. Gene names in bold indicate genes containing top Z Bayenv2.0 SNPs. (PDF 103 kb) [file 12862_2017_929_MOESM12_ESM.pdf]

Additional file 12. List of significantly enriched GO terms for environmentally associated genes. Gene names in bold indicate genes containing top *Z* Bayenv2.0 SNPs.

GO:0051179	**LPUS_01293** **LPUS_01915** LPUS_01919 LPUS_02074 **LPUS_02677** **LPUS_02740** LPUS_02742 **LPUS_02791** **LPUS_02935** **LPUS_02976** **LPUS_03023** **LPUS_03026** **LPUS_03459** LPUS_03487 LPUS_03659 LPUS_03694 LPUS_03949 LPUS_04000 **LPUS_04075** LPUS_04231 LPUS_04290 **LPUS_04297** **LPUS_04453** LPUS_04648 **LPUS_04828** LPUS_04839 **LPUS_04846** **LPUS_05003** **LPUS_05015** **LPUS_05017** **LPUS_05036** LPUS_05069 LPUS_05078 **LPUS_05092** LPUS_05119 LPUS_05242 **LPUS_05296** **LPUS_05319** **LPUS_05476** **LPUS_05516** **LPUS_05533** LPUS_05535 LPUS_05940 **LPUS_05963** **LPUS_06021** LPUS_06047 **LPUS_06065** **LPUS_06114** **LPUS_06132** LPUS_06203 LPUS_06473 LPUS_06672 LPUS_06695 **LPUS_06700** **LPUS_06704** **LPUS_06705** **LPUS_06743** **LPUS_06784** **LPUS_06790** **LPUS_06796** **LPUS_06822** **LPUS_06834** **LPUS_06881** **LPUS_07109** **LPUS_07110** **LPUS_07153** LPUS_07228 LPUS_07296 **LPUS_07352** **LPUS_07452** **LPUS_07509** **LPUS_07552** LPUS_07557 **LPUS_07869** **LPUS_08258** LPUS_08418 **LPUS_08548** **LPUS_08926** **LPUS_08962** **LPUS_08979** **LPUS_09309** **LPUS_09381** **LPUS_09390** LPUS_09393 LPUS_09425 LPUS_09481 **LPUS_09512** LPUS_09615 LPUS_10298 **LPUS_10369** LPUS_10396 LPUS_10446 LPUS_10455 **LPUS_10568** LPUS_10613 **LPUS_10830** LPUS_10831 **LPUS_11004** **LPUS_11287** **LPUS_11315** **LPUS_11330** LPUS_11605 **LPUS_11674** LPUS_11864 LPUS_12375
GO:0007154	LPUS_01993 **LPUS_02512** **LPUS_02733** **LPUS_02812** LPUS_02975 LPUS_02987 LPUS_03198 **LPUS_03892** **LPUS_04075** **LPUS_04828** LPUS_05288 LPUS_05662 LPUS_06047 **LPUS_06504** **LPUS_06582** LPUS_06672 **LPUS_06699** **LPUS_06811** **LPUS_07254** LPUS_07354 **LPUS_07508** **LPUS_08926** **LPUS_09246** LPUS_09385 LPUS_09663 LPUS_10835
GO:0007165	LPUS_01993 **LPUS_02512** **LPUS_02733** LPUS_02975 LPUS_02987 LPUS_03198 **LPUS_03892** **LPUS_04075** **LPUS_04828** LPUS_05288 LPUS_05662 LPUS_06047 **LPUS_06504** **LPUS_06582** LPUS_06672 **LPUS_06811** **LPUS_07254** LPUS_07354 **LPUS_07508** **LPUS_08926** LPUS_09246 LPUS_09663 LPUS_10835
GO:0090305	**LPUS_02236** LPUS_05106 **LPUS_06045** **LPUS_06827** **LPUS_10385** LPUS_11313 LPUS_11863
GO:0006820	**LPUS_01293** **LPUS_01915** LPUS_04000 **LPUS_05036** LPUS_05940 **LPUS_06822** **LPUS_07109** **LPUS_07110** LPUS_07296 LPUS_07557 LPUS_10369 **LPUS_10396** **LPUS_10568** **LPUS_11315**
GO:0006281	**LPUS_01880** **LPUS_03012** **LPUS_03591** **LPUS_04302** **LPUS_04948** **LPUS_05025** LPUS_05105 **LPUS_05508** **LPUS_05593** **LPUS_06045** **LPUS_06121** **LPUS_06731** **LPUS_06827** LPUS_06845 **LPUS_07145** LPUS_07661 LPUS_08921 **LPUS_09521** **LPUS_10430** LPUS_10575 LPUS_10723 **LPUS_10846** LPUS_11021 **LPUS_11313** **LPUS_12124**
GO:0043414	LPUS_02030 LPUS_04372 **LPUS_06035** **LPUS_06802** LPUS_07508 LPUS_07537 LPUS_11303
GO:0009066	**LPUS_00569** **LPUS_01877** LPUS_03969 LPUS_04806 LPUS_05835 LPUS_06658 LPUS_06665 LPUS_08964 LPUS_09416 LPUS_10655
GO:0006553	LPUS_00569 LPUS_04806 LPUS_05835 LPUS_10655
GO:0030258	LPUS_02077 **LPUS_05092** LPUS_05238 **LPUS_05782** **LPUS_06665** **LPUS_06824**
GO:0016226	**LPUS_01889** LPUS_02656 LPUS_04052 LPUS_05288
GO:0031163	**LPUS_01889** LPUS_02656 LPUS_04052 LPUS_05288
GO:0008213	**LPUS_06035** LPUS_07508 LPUS_07537
GO:0006479	**LPUS_06035** LPUS_07508 LPUS_07537
GO:0046834	**LPUS_05238** **LPUS_06665** **LPUS_06824**
GO:0046854	**LPUS_05238** **LPUS_06665** **LPUS_06824**
GO:0006614	**LPUS_06704** **LPUS_09512** LPUS_11287 LPUS_12375
GO:0030259	LPUS_**02077** **LPUS_05092** **LPUS_05782**
GO:0016571	**LPUS_06035** LPUS_07508 LPUS_07537
GO:0042176	**LPUS_04834** LPUS_05589 LPUS_06835 LPUS_11027
GO:0006273	LPUS_06827 LPUS_08313 LPUS_10575
GO:0008608	**LPUS_02976** **LPUS_06834** **LPUS_06881**
GO:0070592	**LPUS_01977** LPUS_05092 LPUS_09679
