# Supplementary material for: Adaptive differentiation coincides with local bioclimatic conditions along an elevational cline in populations of a lichen-forming fungus
Source: BMC Evol Biol. 2017 Mar 31;17:93. doi: 10.1186/s12862-017-0929-8 (PMC5374679; doi:10.1186/s12862-017-0929-8)

Additional file 13. Neighbor-joining tree of the aligned concatenated gene fragments of the six loci at which we genotyped 18 thalli *L. pustulata* used for the anatomical and ecophysiological measurements. Samples in bold and underlined (N = 6) were used for the gas exchange experiments.

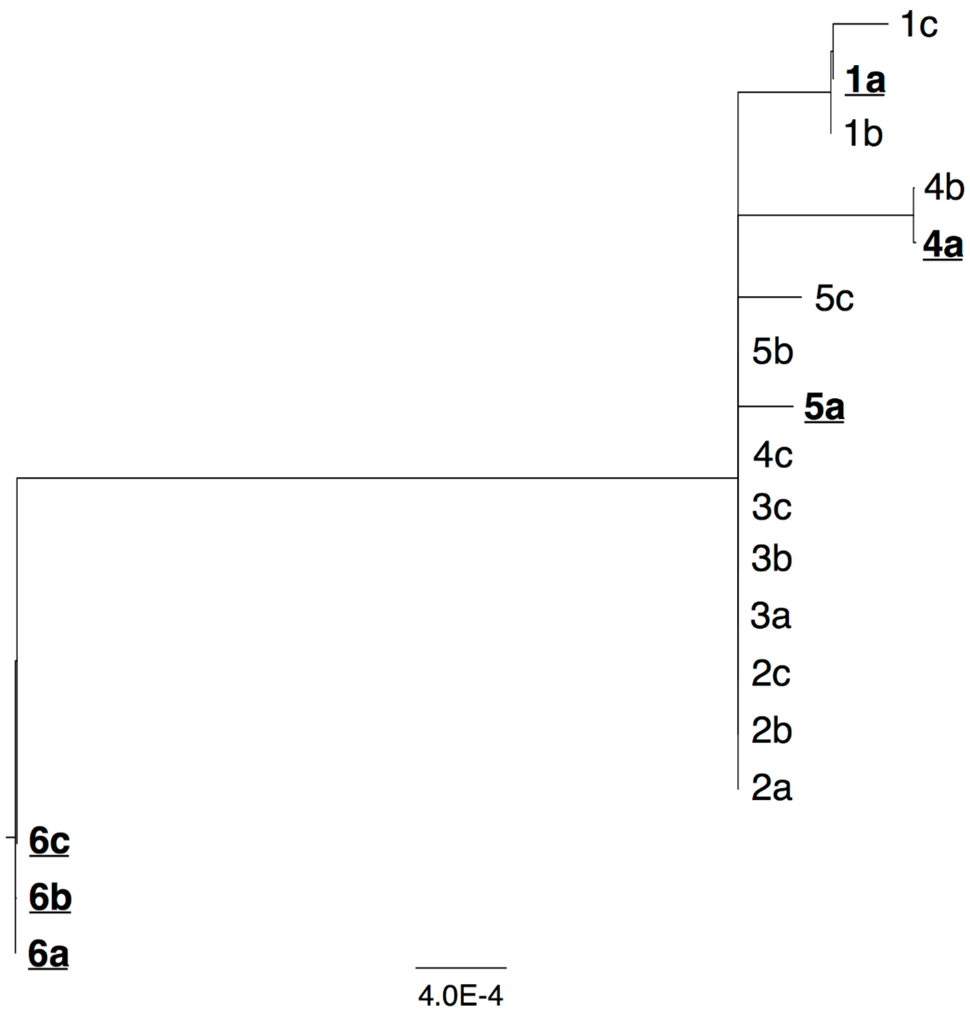

Supplement: Supplementary file 13 — Neighbor-joining tree of the aligned concatenated gene fragments of the six loci at which we genotyped 18 thalli L. pustulata used for the anatomical and ecophysiological measurements. Samples in bold and underlined (N = 6) were used for the gas exchange experiments. (PDF 165 kb) [file 12862_2017_929_MOESM13_ESM.pdf]
