# Supplementary material for: Adaptive differentiation coincides with local bioclimatic conditions along an elevational cline in populations of a lichen-forming fungus
Source: BMC Evol Biol. 2017 Mar 31;17:93. doi: 10.1186/s12862-017-0929-8 (PMC5374679; doi:10.1186/s12862-017-0929-8)

Additional file 14. Light (a), temperature (b) curves for 6 thalli representing two genetic groups (1: pop. 6, high altitude; 2: populations 1 to 5, low altitude).

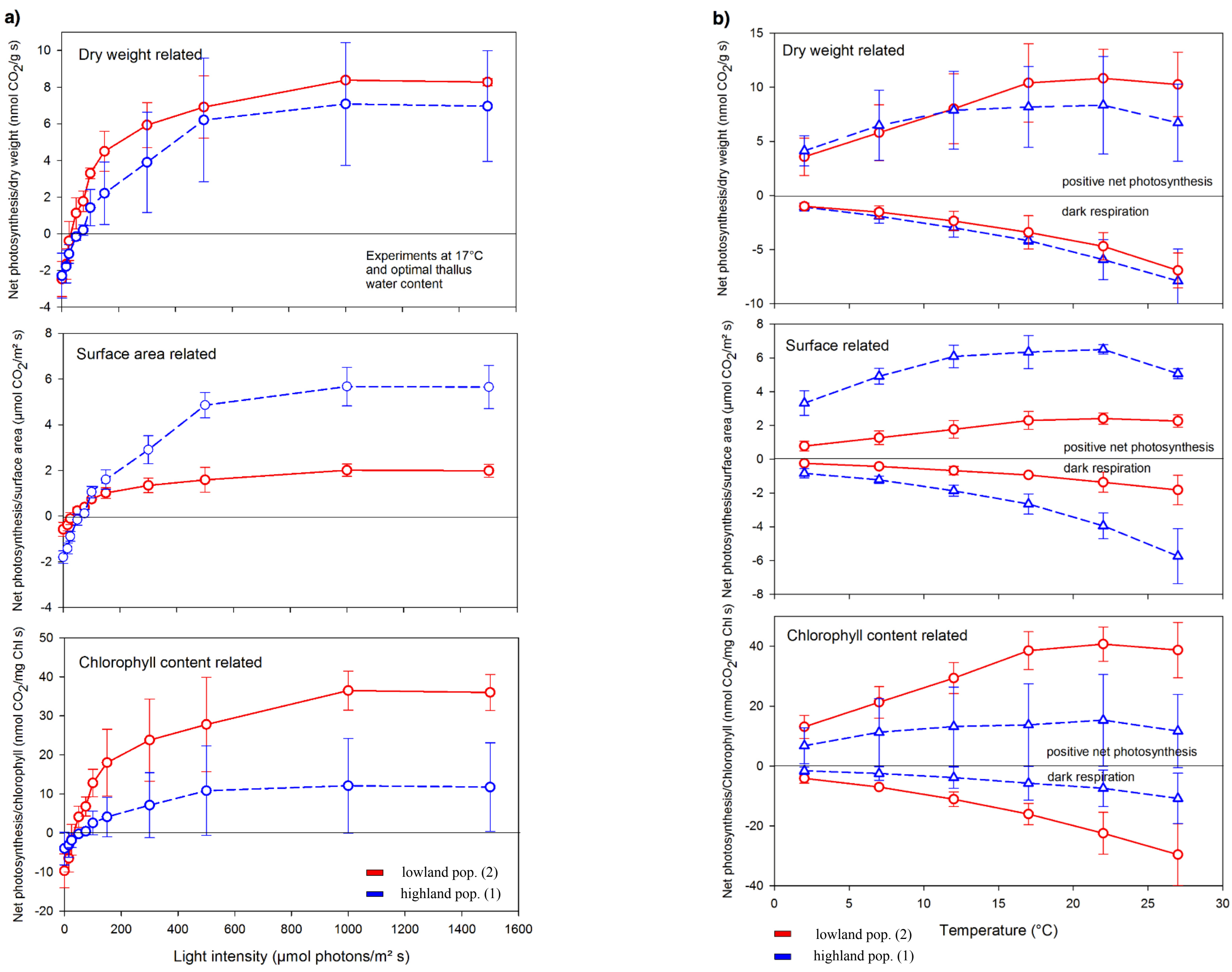

Supplement: Supplementary file 14 — Light (a), temperature (b) curves for 6 thalli representing two genetic groups (1: pop. 6, high altitude; 2: populations 1 to 5, low altitude). (PDF 556 kb) [file 12862_2017_929_MOESM14_ESM.pdf]
