# Supplementary material for: Adaptive differentiation coincides with local bioclimatic conditions along an elevational cline in populations of a lichen-forming fungus
Source: BMC Evol Biol. 2017 Mar 31;17:93. doi: 10.1186/s12862-017-0929-8 (PMC5374679; doi:10.1186/s12862-017-0929-8)
Supplement: Supplementary file 15 — Net photosynthesis at optimal thallus water content and optimal temperature for genetic group A (pop. 6, high altitude) and B (populations 1 to 5, low altitude). (PDF 94 kb) [file 12862_2017_929_MOESM15_ESM.pdf]

Additional file 15. Net photosynthesis at optimal thallus water content and optimal temperature for genetic group A (pop. 6, high altitude) and B (populations 1 to 5, low altitude).

	Population	90% of maximal net photosynthesis	at [µmol photons/m · s]	maximal net photosynthesis	at [µmol photons/m · s]
thallus dry weight	A (high)	6.36 nmol CO_2/g · s	580	7.07	1000
	B (low)	7.56 nmol CO_2/g · s	740	8.4	1000
thallus surface area	A (high)	5.41 µmol CO_2/m · s	717	5.71	1000
	B (low)	1.8 µmol CO_2/m · s	727	2.0	1000
chlorophyll *a+b*	A (high)	10.76 nmol CO_2/mg Chl · s	500	11.95	1000
	B (low)	32.79 nmol CO_2/mg Chl · s	780	36.43	1000
